# An integrated single-cell analysis of human adrenal cortex development

**DOI:** 10.1172/jci.insight.168177

**Published:** 2023-07-24

**Authors:** Ignacio del Valle, Matthew D. Young, Gerda Kildisiute, Olumide K. Ogunbiyi, Federica Buonocore, Ian C. Simcock, Eleonora Khabirova, Berta Crespo, Nadjeda Moreno, Tony Brooks, Paola Niola, Katherine Swarbrick, Jenifer P. Suntharalingham, Sinead M. McGlacken-Byrne, Owen J. Arthurs, Sam Behjati, John C. Achermann

**Affiliations:** 1 Genetics and Genomic Medicine Research and Teaching Department, University College London (UCL) Great Ormond Street Institute of Child Health, UCL, London, United Kingdom.; 2 Wellcome Sanger Institute, Wellcome Genome Campus, Hinxton, United Kingdom.; 3 Department of Histopathology, Great Ormond Street Hospital for Children National Health Service (NHS) Foundation Trust, London, United Kingdom.; 4 Developmental Biology and Cancer Research and Teaching Department, UCL Great Ormond Street Institute of Child Health, UCL, London, United Kingdom.; 5 Department of Clinical Radiology, Great Ormond Street Hospital for Children NHS Foundation Trust, London, United Kingdom.; 6 National Institute of Health Research (NIHR) Great Ormond Street Biomedical Research Centre, London, United Kingdom.; 7 Population, Policy and Practice Research and Teaching Department, UCL Great Ormond Street Institute of Child Health, UCL, London, United Kingdom.; 8 UCL Genomics, Zayed Centre for Research, UCL Great Ormond Street Institute of Child Health, UCL, London, United Kingdom.; 9 Cambridge University Hospitals NHS Foundation Trust, Cambridge, United Kingdom.; 10 Department of Paediatrics, University of Cambridge, Cambridge, United Kingdom.

**Keywords:** Development, Endocrinology, Embryonic development, Genetic diseases, Transcription

## Abstract

The adrenal glands synthesize and release essential steroid hormones such as cortisol and aldosterone, but many aspects of human adrenal gland development are not well understood. Here, we combined single-cell and bulk RNA sequencing, spatial transcriptomics, IHC, and micro-focus computed tomography to investigate key aspects of adrenal development in the first 20 weeks of gestation. We demonstrate rapid adrenal growth and vascularization, with more cell division in the outer definitive zone (DZ). Steroidogenic pathways favored androgen synthesis in the central fetal zone, but DZ capacity to synthesize cortisol and aldosterone developed with time. Core transcriptional regulators were identified, with localized expression of HOPX (also known as Hop homeobox/homeobox-only protein) in the DZ. Potential ligand-receptor interactions between mesenchyme and adrenal cortex were seen (e.g., *RSPO3*/*LGR4*). Growth-promoting imprinted genes were enriched in the developing cortex (e.g., *IGF2*, *PEG3*). These findings reveal aspects of human adrenal development and have clinical implications for understanding primary adrenal insufficiency and related postnatal adrenal disorders, such as adrenal tumor development, steroid disorders, and neonatal stress.

## Graphical abstract

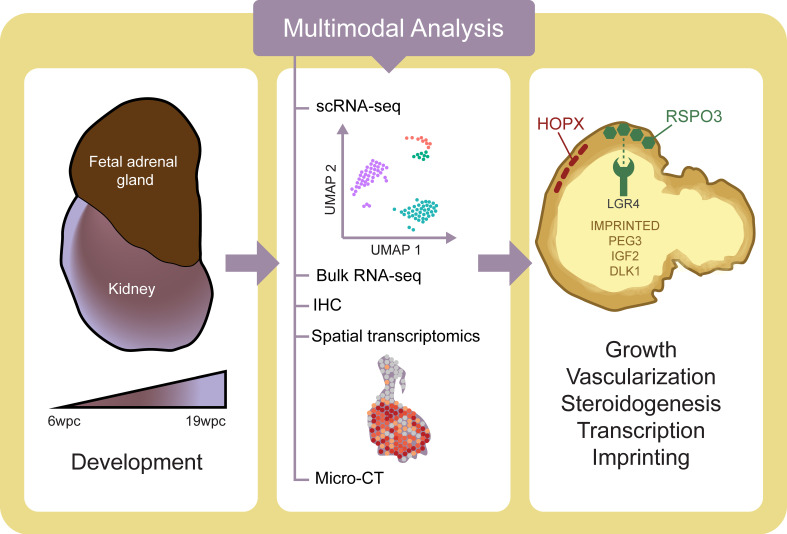

## Introduction

The mature adult adrenal glands are essential endocrine organs that consist of an outer cortex and a central medulla. The adrenal cortex has 3 layers that synthesize and release key groups of steroid hormones (1–4). Mineralocorticoids (e.g., aldosterone) are released from the outer zona glomerulosa and are needed for salt retention and blood pressure maintenance. Glucocorticoids (e.g., cortisol) are released predominantly from the zona fasciculata and are needed for well-being and glucose regulation. Weak androgens (e.g., dehydroepiandrosterone) are released from the inner zona reticularis and influence adrenarche in midchildhood, with potential effects on health in adult women (5–7). In contrast, the central adrenal medulla is neuroectodermal in origin and releases epinephrine (adrenaline) and norepinephrine (noradrenaline) (8). Thus, the adrenal glands play an essential role in the acute stress response, many aspects of physiological homeostasis, and long-term well-being.

Disruption of adrenal gland function (known as primary adrenal insufficiency, PAI) leads to glucocorticoid insufficiency, often combined with mineralocorticoid insufficiency (9, 10). PAI can present at various ages with symptoms such as malaise, weight loss, hyperpigmentation, and hypotension and can be fatal if not diagnosed and treated appropriately (9). Although autoimmune destruction of the adrenal gland (sometimes referred to as Addison’s disease) is the most common cause of PAI in adolescents and adults, around 30 single-gene disorders have now been identified that result in PAI through diverse processes, such as impaired development (hypoplasia), blocks in steroid biosynthesis (congenital adrenal hyperplasia, CAH), adrenocorticotropic hormone (ACTH) resistance (familial glucocorticoid deficiency), and metabolic conditions (10–12). PAI often presents soon after birth or more gradually in childhood or even adulthood. Individuals with PAI require lifelong adrenal steroid hormone replacement, with management sometimes modified based on the underlying cause (9, 13).

In humans, the adrenal cortex develops from bilateral thickenings of the coelomic epithelium (posterior intermediate mesoderm, “adrenogonadal primordium”), and a distinct adrenal primordium forms by 5 weeks postconception (wpc) (7 weeks’ gestation) (Carnegie stage [CS] 15/16) (14–16). The adrenal cortex and gonad share several distinct functional pathways, such as the ability to synthesize steroid hormones and regulation by the nuclear receptor, NR5A1 (also known as steroidogenic factor-1, SF-1) (17–20). In contrast, the adrenal medulla is ectodermal in origin and arises from Schwann cell precursor cells that migrate into the adrenal gland and differentiate into sympathoblastic and chromaffin cells (8, 21). These cells ultimately coalesce centrally to form the adrenal medulla.

Although insights into adrenal development and function are being obtained from studies in model systems (e.g., mice, zebrafish) (22–25), the adrenal cortex in humans and higher primates has distinct structural and functional components (26). Most notable is the development of a large fetal zone (FZ), which is capable of synthesizing and releasing substantial amounts of the weak androgen, dehydroepiandrosterone (DHEA) and its sulfated form, DHEA-S. DHEA is converted to estrogens by the placenta, which enter the maternal circulation during pregnancy (15). The FZ regresses in the first few months of postnatal life (26, 27). The teleological function of the FZ is not known, though DHEA may have a role in neurodevelopment (28). Mice have an X zone that regresses with sexual maturity (males) or pregnancy (females), but similarities with the human FZ are somewhat limited (29–31). Furthermore, cortisol is the primary glucocorticoid synthesized by the adrenal gland in humans whereas rodents generate higher concentrations of corticosterone (3).

In recent years, a limited number of studies of human adrenal development or fetal adrenal steroidogenesis have been undertaken using gene expression approaches or focused reverse transcription PCR/IHC of steroid pathways (17, 32–36). More recently, several studies have started to address human adrenal development or steroidogenesis using single-cell or spatial transcriptomics (16, 37–39), and induced pluripotent stem cells (iPSCs) have been explored as a model of human fetal adrenal development (40). However, few data currently exist for detailed transcriptomic analysis of the human adrenal cortex at a single-cell level during the critical first half of gestation (to 20wpc) or transcriptomics linked to developmental anatomy. We therefore developed a multimodal approach to investigate human adrenal cortex development in detail.

## Results

### The developing adrenal gland has a defined transcriptomic profile.

**Figure 1:**
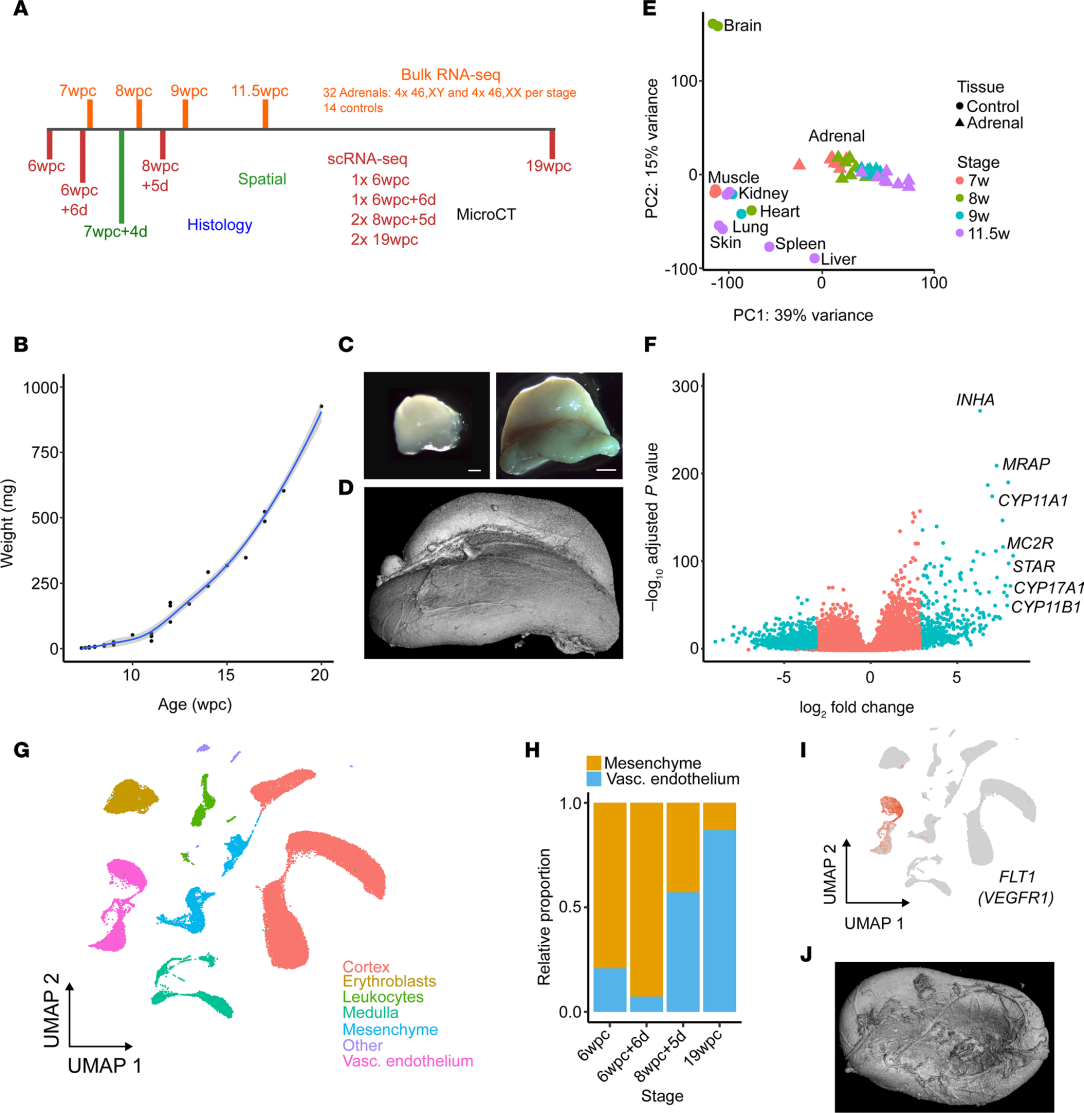
Study design, adrenal development, and transcriptome analysis. (**A**) Overview of the study design for generating bulk transcriptomes (bulk RNA-Seq), single-cell mRNA transcriptomes (scRNA-Seq), spatial transcriptomics, micro-CT (micro-focus computed tomography), and histology/immunohistochemistry. Stages are shown as weeks (w) and days (d) postconception (pc). (**B**) Growth curve of the adrenal gland between 7 weeks postconception and 2 days (7wpc+2d) and 20wpc (*n* = 36). Data for single glands are shown. (**C**) Photographs of adrenal glands (10% formalin) at 6wpc+6d (left, scale bar: 300 μm) and 16wpc (right, scale bar: 3 mm) to show marked growth and anatomical changes. (**D**) Micro-CT surface image of the adrenal gland at 17wpc showing the anterior sulcus and vascularization (maximum dimension, 15 mm). (**E**) Principal component analysis (PCA) of bulk transcriptome data for adrenal glands at 7wpc (*n* = 8), 8wpc (*n* = 8), 9wpc (*n* = 8), and 11.5wpc (*n* = 8) and control tissues (*n* = 14, from 8 different tissues) as indicated. (**F**) Volcano plot showing differential gene expression of genes in the bulk transcriptome adrenal gland data set (total *n* = 32) compared with controls (*n* = 14). Selected highly differentially expressed adrenal genes are indicated. (**G**) Uniform manifold approximation and projection (UMAP) of scRNA-Seq transcriptome data from 4 adrenal glands (6w, 6wpc+6d, 8wpc+5d, 19w) with the major different cell populations annotated (6wpc, *n* = 3,047 cells; 6wpc+6d, *n* = 2,650 cells; 8wpc+5d, *n* = 23,313 cells; 19wpc, *n* = 15,348 cells). (**H**) Relative proportion of mesenchyme and vascular endothelial cells in the adrenal gland at each time point studied. (**I**) Feature plot showing expression of *FLT1* (encoding vascular endothelial growth factor receptor 1, VEGFR1) in the vascular endothelial cluster (see **G** for annotation). (**J**) Micro-CT (17wpc) to show the extensive surface vascular network on the inferior surface of the gland (maximum dimension, 15 mm).

To study the key biological events involved in human adrenal development, we integrated single-cell RNA sequencing (scRNA-Seq), bulk RNA-Seq, spatial transcriptomics, IHC, and micro-focused computed tomography (micro-CT) imaging across a critical developmental time frame between 6wpc and 20wpc (Figure 1A and Supplemental Data 1; supplemental material available online with this article; https://doi.org/10.1172/jci.insight.168177DS1). During this period, the adrenal gland undergoes rapid growth and specific morphological changes such as the development of a deep sulcus (Figure 1, B–D; Supplemental Figure 1; and Supplemental Video 1).

At a global level (bulk RNA-Seq), the developing adrenal gland showed a well-defined transcriptomic profile compared with control tissues. This transcriptome was more similar to mesodermal structures (e.g., kidney, heart, muscle) at early stages of development but became increasingly distinct with age (Figure 1E and Supplemental Figure 2). A subset of highly differentially expressed adrenal specific genes was identified, including known genes — e.g., ACTH receptor (*MC2R*), steroidogenic acute regulator (*STAR*), and *CYP11A1* (encoding the P450 side-chain cleavage enzyme) — as well as several genes not previously identified as differentially expressed in adrenal development, e.g., *CLRN1*, *MIR202HG*, and *FAM166B* (Figure 1F, Supplemental Figure 2, and Supplemental Data 2).

**Figure 2:**
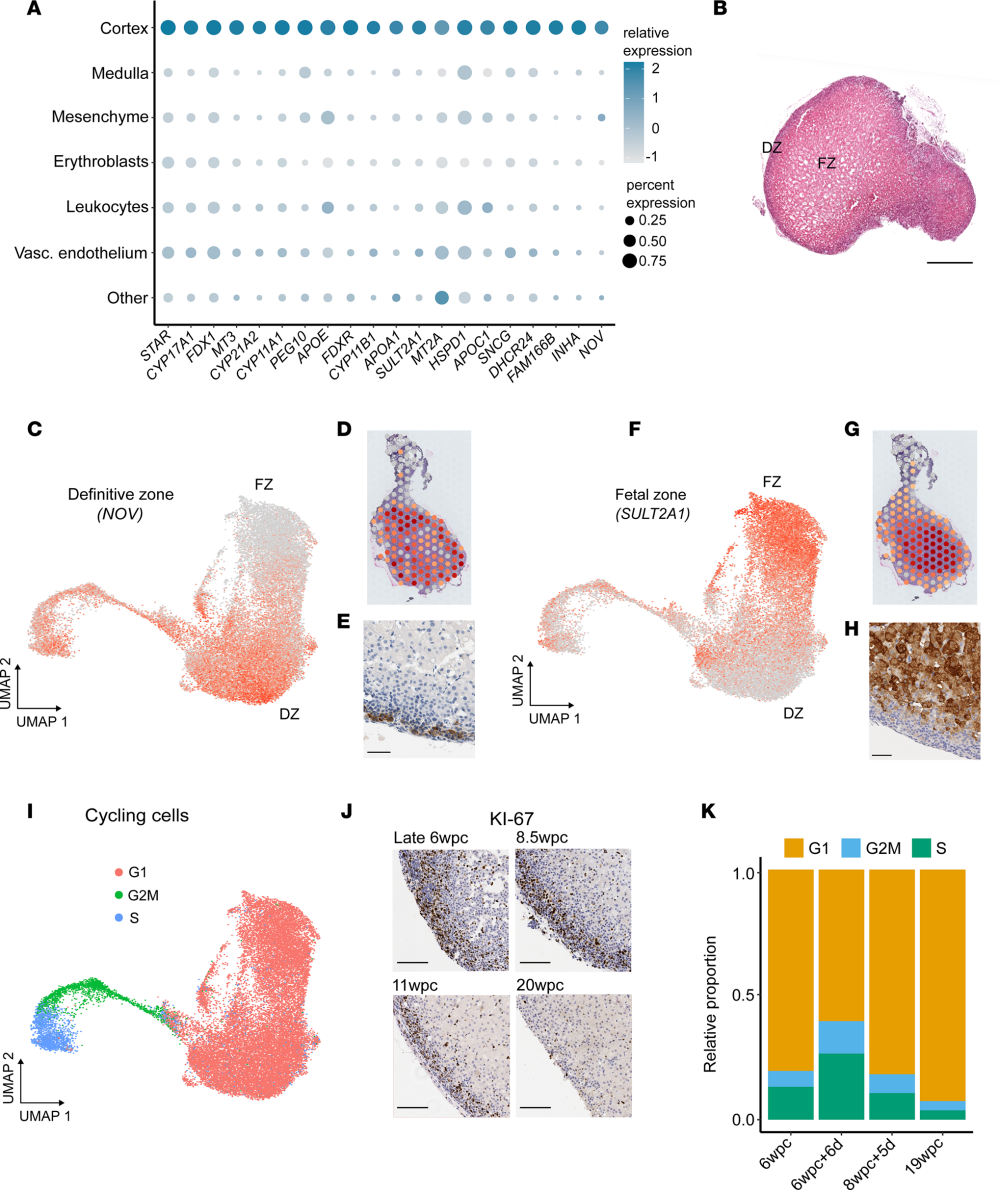
Adrenal cortex zonation and proliferation. (**A**) Dot plot to show the most highly differentially expressed genes in the adrenal cortex single-cell transcriptome (scRNA-Seq) compared with other cells in the adrenal gland. (**B**) Histology of the human fetal adrenal gland at 8.5wpc (H&E staining). Scale: 400 μm. DZ, definitive zone; FZ, fetal zone. (**C**–**E**) The developing DZ shown by *NOV* (also known as *CCN3*) expression using a single-cell mRNA transcriptome UMAP (**C**), spatial transcriptomic spot plot (7wpc+4d, darker red shows higher expression) (**D**) and immunohistochemistry (11wpc; scale: 50 μm) (**E**). Integrated data from samples at all 4 time points are shown. (**F**–**H**) The developing FZ shown by *SULT2A1* expression using a single-cell mRNA transcriptome UMAP (**F**), spatial transcriptomic spot plot (7wpc+4d) (**G**) and immunohistochemistry (11wpc; scale: 50 μm) (**H**). (**I**) Integrated UMAP showing cell cycle states. (**J**) IHC of fetal adrenal gland showing Ki-67 expression as a marker of cell proliferation at different ages. Scales: all 100 μm. (**K**) Relative proportion of cells in each cell cycle state at each time point.

To define specific cell populations within the adrenal gland in more detail, single-cell mRNA transcriptome analysis (scRNA-Seq) was undertaken at 4 time points (6wpc, 6wpc+6days [d], 8wpc+5d, 19wpc) (Figure 1, A and G) (21). This analysis clearly identified a cluster of adrenal cortex cells, with strong enrichment for genes involved in steroidogenesis (Figure 1G, Figure 2A, and Supplemental Data 3). Other major clusters included cells that contribute to the developing adrenal medulla (Schwann cell precursors, sympathoblastic cells, chromaffin cells, and recently described “medullary bridge” cells) (21), as well as mesenchymal cells, vascular endothelial cells, erythroblasts, and leukocytes (Figure 1G and Supplemental Figure 3A). The relative proportion of mesenchymal cells decreased over time with differentiation, whereas the vascular endothelial components and erythroblast cells increased (Figure 1H and Supplemental Figure 3). Marked differential expression of *FLT1* (encoding vascular endothelial growth factor receptor 1, VEGFR1) was seen within the vascular endothelial cluster, which increased with time (Figure 1I, Supplemental Figure 3, and Supplemental Data 4). These findings are consistent with the development of an extensive vascular network supplying the adrenal gland and a network of sinusoids within it, necessary for the release of steroid hormones into the developing fetal circulation (Figure 1, H–J, and Supplemental Figure 4).

### The adrenal cortex has distinct zones.

Subsequent analysis focused on the fetal adrenal cortex (Figure 1G, Figure 2A, and Supplemental Figure 5), as relatively few data are available for cortex development in humans, especially in the second trimester, and single-cell mRNA transcriptome analysis allows new insights to be obtained.

Histologically, the fetal adrenal cortex is broadly divided into an outer definitive zone (DZ), somewhat similar to the postnatal zona glomerulosa and zona fasciculata, and an inner FZ consisting of large cytomegalic cells interspersed with vascular sinusoids (Figure 2B and Supplemental Figure 4). A distinct capsule forms around the adrenal gland during the first trimester, with a putative transition zone developing later in the second trimester (26).

To study cortex zonation in more detail, we used nephroblastoma overexpressed/cysteine-rich protein 61/connective tissue growth factor/nephroblastoma overexpressed gene-3 (*NOV*, also known as *CCN3*) as a marker for the DZ and sulfotransferase 2A1 (*SULT2A1*) as a marker for the FZ (35, 41, 42). These genes differentiated the DZ and FZ clearly in an integrated scRNA-Seq data set, as well as by spatial transcriptomics (7wpc+4d) and IHC (11wpc data shown) (Figure 2, C–H). Of note, more DZ cells were cycling (S phase, G2M phase) compared with FZ cells (Figure 2I and Supplemental Figure 6). This finding was supported by IHC using Ki-67 as a marker of cell division (Figure 2J). The relative proportion of dividing cells was highest during early development (Figure 2, J and K), consistent with rapid growth of the gland during this time (Figure 1C and Supplemental Figure 1). During the earliest stage (6w), a trajectory of cells from the DZ to FZ was seen (Supplemental Figure 6D). Taken together, these data suggest that the DZ is a more active region of cell division compared with the FZ, and with potential centripetal cell differentiation at least in early development (43).

### Fetal adrenal steroidogenesis favors DHEA synthesis.

A major role of the mature, postnatal adrenal cortex is to synthesize and release steroid hormones, such as cortisol and aldosterone. The extent to which the developing adrenal gland has the biosynthetic capacity to produce these steroids is still unclear. It is well established that the FZ synthesizes and releases large amounts of DHEA(-S), due to a relative lack of the enzyme 3 β-hydroxysteroid dehydrogenase type 2 (3β-HSD2, encoded by *HSD3B2*) and likely high expression of genes encoding enzymes needed for androgen biosynthesis (i.e., *CYP17A1*, *POR*, *CYB5A*). Although a transient wave of *HSD3B2*/3β-HSD2 expression has been shown at around 8–9wpc (33, 35, 39), evidence is still emerging as to when the necessary enzymes for glucocorticoid (e.g., cortisol) and mineralocorticoid (e.g., aldosterone) synthesis are expressed during human adrenal development, especially into the second trimester (34, 35).

**Figure 3:**
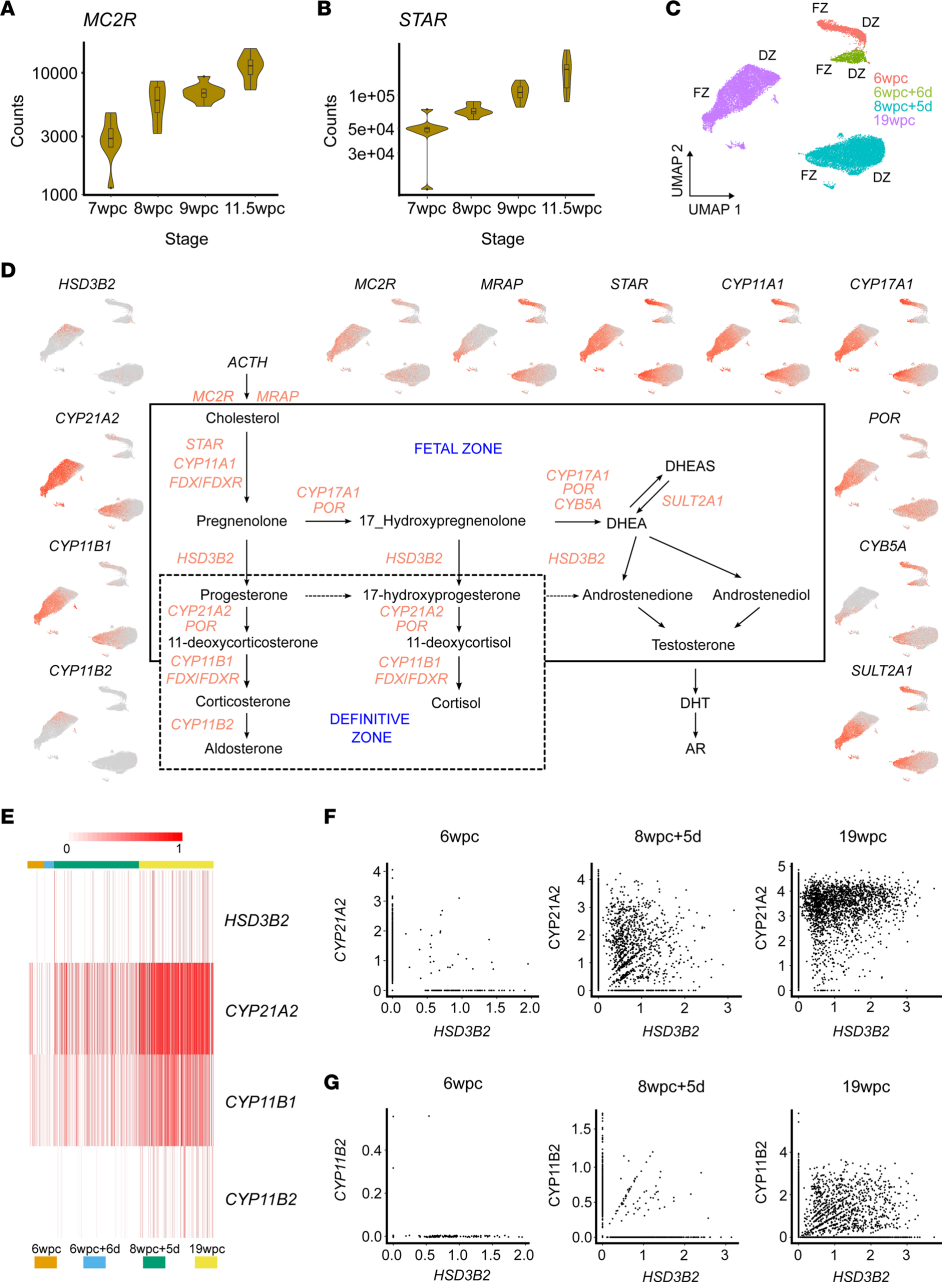
Expression of classic steroidogenic pathway genes during human adrenal development. (**A**) Time series bulk RNA-Seq expression (normalized counts) of the melanocortin-2 receptor gene (*MC2R*), encoding the adrenocorticotropin (ACTH) receptor (*n* = 8 at each stage). Violin plots show the median values (horizontal bars), outliers, and the distribution of upper and lower interquartile ranges (boxes). (**B**) Time series bulk RNA-Seq expression of the gene encoding steroidogenic acute regulatory protein (*STAR*) (*n* = 8 at each stage). (**C**) UMAP of adrenal cortex clusters used for subsequent analysis. DZ, definitive zone; FZ, fetal zone. (**D**) Graphical representation of the “classic” steroidogenic pathway showing the key genetic components leading to the synthesis of mineralocorticoids (e.g., aldosterone), glucocorticoids (e.g., cortisol), and androgens (e.g., DHEA, androstenedione, testosterone). Feature plots showing the expression of key genes in the adrenal cortex clusters at different time points are shown. ACTH, adrenocorticotropin; AR, androgen receptor; *CYB5A*, cytochrome 5A; *CYP11A1*, P450 side-chain cleavage enzyme; *CYP11B1*, 11β-hydroxylase type 1; *CYP11B2*, aldosterone synthase; *CYP17A1*, 17α-hydroxylase/17,20-lyase; *CYP21A2*, 21-hydroxylase; DHEA(S), dehydroepiandrosterone (sulfate); DHT, dihydrotestosterone; *HSD3B2*, 3β-hydroxysteroid dehydrogenase type 2; *MC2R*, melanocortin-2 receptor (ACTHR); *MRAP*, MC2R accessory protein; *POR*, P450 oxidoreductase. (**E**) Heatmap of scRNA-Seq expression of *HSD3B2*, *CYP21A2*, *CYP11B1*, and *CYP11B2* at different ages in the adrenal cortex clusters. (**F**) Scatterplots of expression of *HSD3B2* in individual cortex single cells (scRNA-Seq) compared with *CYP21A2* at 3 different time points (6wpc, 8wpc+5d, 19wpc). (**G**) Scatterplots of expression of *HSD3B2* in individual cortex single cells (scRNA-Seq) compared with *CYP11B2* at 3 different time points (6wpc, 8wpc+5d, 19wpc).

To explore this further, we analyzed time series bulk RNA-Seq data (between 7wpc and 11.5wpc), which showed a clear temporal increase in expression of *MC2R*, as well as *STAR* and most other steroidogenic enzymes (Figure 3, A and B, and Supplemental Figure 7). These data show that the machinery for ACTH-dependent cholesterol processing is in place during early development and increases with age.

Next, a scRNA-Seq data set was generated subsetting the annotated adrenal cortex cells at each time point studied, with cycling cells removed (see UMAP, Figure 3C). Across all stages, cells of the FZ region showed high expression of genes encoding the key enzymes needed for DHEA synthesis (*STAR*, *CYP11A1*, *CYP17A1*, *POR*, *CYB5A*) as well as of *SULT2A1*, which is required for sulfation of DHEA to DHEA-S and protects the developing fetus from androgen exposure (Figure 3D). As expected, *HSD3B2* expression was low in the FZ during development, resulting in the likely shuttling of steroid precursors (e.g., pregnenolone) into the androgen pathway. The high expression of *STAR* and *CYP11A1* in the FZ cluster was mirrored by high expression of the ACTH receptor and its accessory protein (*MRAP*), suggesting not only that the FZ is biosynthetically active but also that FZ DHEA synthesis may have the capacity to be ACTH dependent. Of note, enzymes proposed to be involved in the “backdoor” pathway of androgen synthesis (36) were not strongly expressed, although several components of the pathway needed for 11-oxygenation of androgens (44) were (Supplemental Figures 8–10).

As 3β-hydroxysteroid dehydrogenase type 2/*HSD3B2* is effectively a gatekeeper to glucocorticoid and mineralocorticoid biosynthesis (Figure 3D), we investigated *HSD3B2* expression across time series data. Although a potential transiently higher expression was seen at 8wpc in bulk RNA-Seq data (Supplemental Figure 7), as reported previously (33, 35, 39), single-cell transcriptomic data showed overall greater increase in *HSD3B2* across time, with the highest levels in the DZ cluster at 19wpc (Figure 3E). A similar graded increase in *CYP21A2* (encoding 21-hydroxylase) and *CYP11B1* (encoding 11β-hydroxylase type 1) was seen (Figure 3E). Single-cell gene coexpression analysis revealed a distinct subset of cells that coexpressed *HSD3B2* and *CYP21A2* by 8wpc+5d, although by 19wpc it appeared that *CYP21A2* expression occurred in a greater number of cells and that expression of *HSD3B2* (and its protein) was the likely rate-limiting factor (Figure 3F). These likely represent transitional zone cells in the DZ. Expression of *CYP11B1* also increased from 8wpc+5d, especially in *CYP21A2*^+^ cells (Supplemental Figure 11). Taken together, these data suggest that there is an increase in gene expression of the enzymatic machinery needed for glucocorticoid synthesis across time. The specific transcriptional regulators leading to increased *HSD3B2* expression are not known, although based on previous candidates our data suggest it is likely NR5A1, NR4A1 (NURR77), and GATA6 contribute (Supplemental Figure 12) (45).

It is also debated at what stage the developing fetal adrenal gland can synthesize mineralocorticoids, such as aldosterone (34, 35). Here, *CYP11B2* (encoding 11β-hydroxylase type 2/aldosterone synthase) is a key enzyme in the final stages of aldosterone synthesis, as well as *HSD3B2*, which is needed to allow precursors into this pathway (Figure 3D). In our scRNA-Seq data, *CYP11B2* expression was low in early stages but increased by 19wpc in a developing zona glomerulosa (zG) subpopulation of cells in the DZ (Figure 3, D, E, and G).

### Transcriptional regulation of the fetal adrenal cortex.

**Figure 4:**
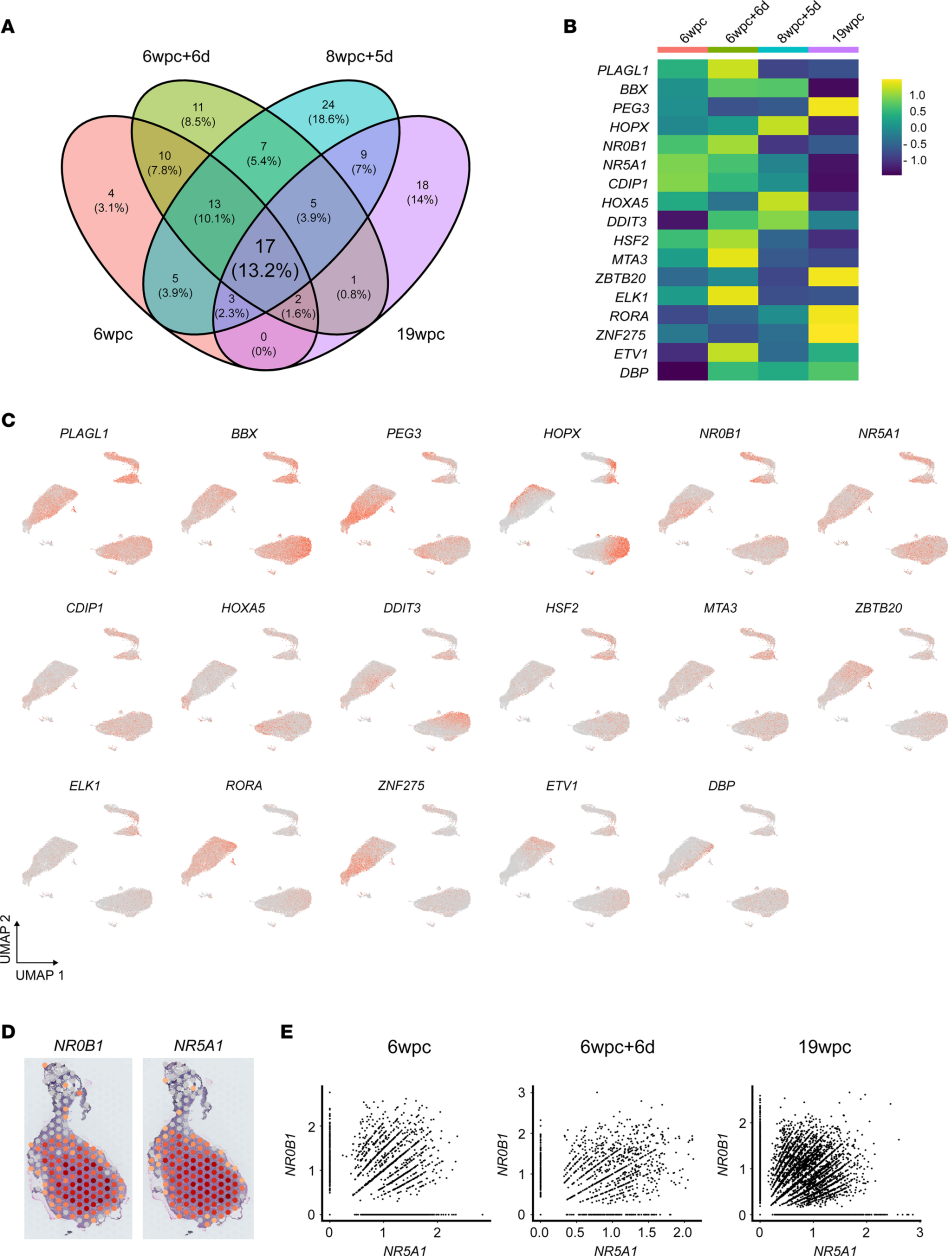
Expression of transcription factors during human adrenal cortex development. (**A**) Venn diagram showing the overlap of differentially expressed transcription factors in the scRNA-Seq data set at each age. Differential expression was defined as being enriched in the adrenal cortex cluster compared with all other clusters in the whole adrenal sample at each age (log_2_FC > 0.25, padj < 0.05). A core group of 17 transcription factors common to all ages was identified. (**B**) Heatmap showing relative expression of these 17 transcription factors at each age in the scRNA-Seq data set. (**C**) Feature plots showing expression of these 17 transcription factors in adrenal cortex clusters (for annotation, see Figure 3C). (**D**) Spatial transcriptomic spot plot expression of the key nuclear receptors, *NR0B1* (also known as DAX-1) and *NR5A1* (also known as steroidogenic factor-1, SF-1) at 7wpc+4d. (**E**) Scatterplots of expression of *NR5A1* in individual adrenal cortex single cells (scRNA-Seq) compared with *NR0B1* (6wpc, 6wpc+5d, 19wpc).

To study “core” transcriptional regulators of human adrenal cortex, we first identified genes that were differentially expressed in the cortex cluster compared with noncortex clusters at each scRNA-Seq stage (log_2_ fold-change [log_2_FC] > 0.25, adjusted *P* [padj] < 0.05) and compared these genes to the Animal Transcription Factor Database (46) (Supplemental Data 5). At each developmental time point studied, transcription factors represented between 1.8% and 2.4% of all differentially expressed cortex genes (Supplemental Figure 13A). By intersecting these analyses, 17 “core” transcriptional regulators were identified that were common to all data sets (Figure 4, A–C, and Supplemental Figures 13 and 14). These factors were also present in bulk RNA-Seq analysis of adrenal gland samples compared with control tissues (padj < 0.05) (Supplemental Figure 13, B and C, and Supplemental Data files 2 and 5).

Two key transcription factors that are well-established regulators of adrenal development are the orphan nuclear receptors, NR0B1 (DAX-1) and NR5A1 (SF-1) (14, 17, 19, 20). Disruption of NR0B1 causes X-linked adrenal hypoplasia, which is one of the most common causes of PAI in children (boys) (12, 47). NR5A1 is a master regulator of adrenal and reproductive development and function, and more severe disruption is also associated with PAI in humans (20, 48). Several studies have suggested that NR0B1 and NR5A1 can be functional partners, but data about expression in human development are still limited (14, 17, 29, 49). Cluster analysis in scRNA-Seq data sets as well as spatial transcriptomic analysis showed that expression of *NR0B1* and *NR5A1* occurred extensively throughout the fetal adrenal gland, especially in the FZ (Figure 4, C–E). Taken together, these data demonstrate the importance of NR0B1 and NR5A1 in human adrenal development.

### HOPX is a potentially novel DZ factor.

**Figure 5:**
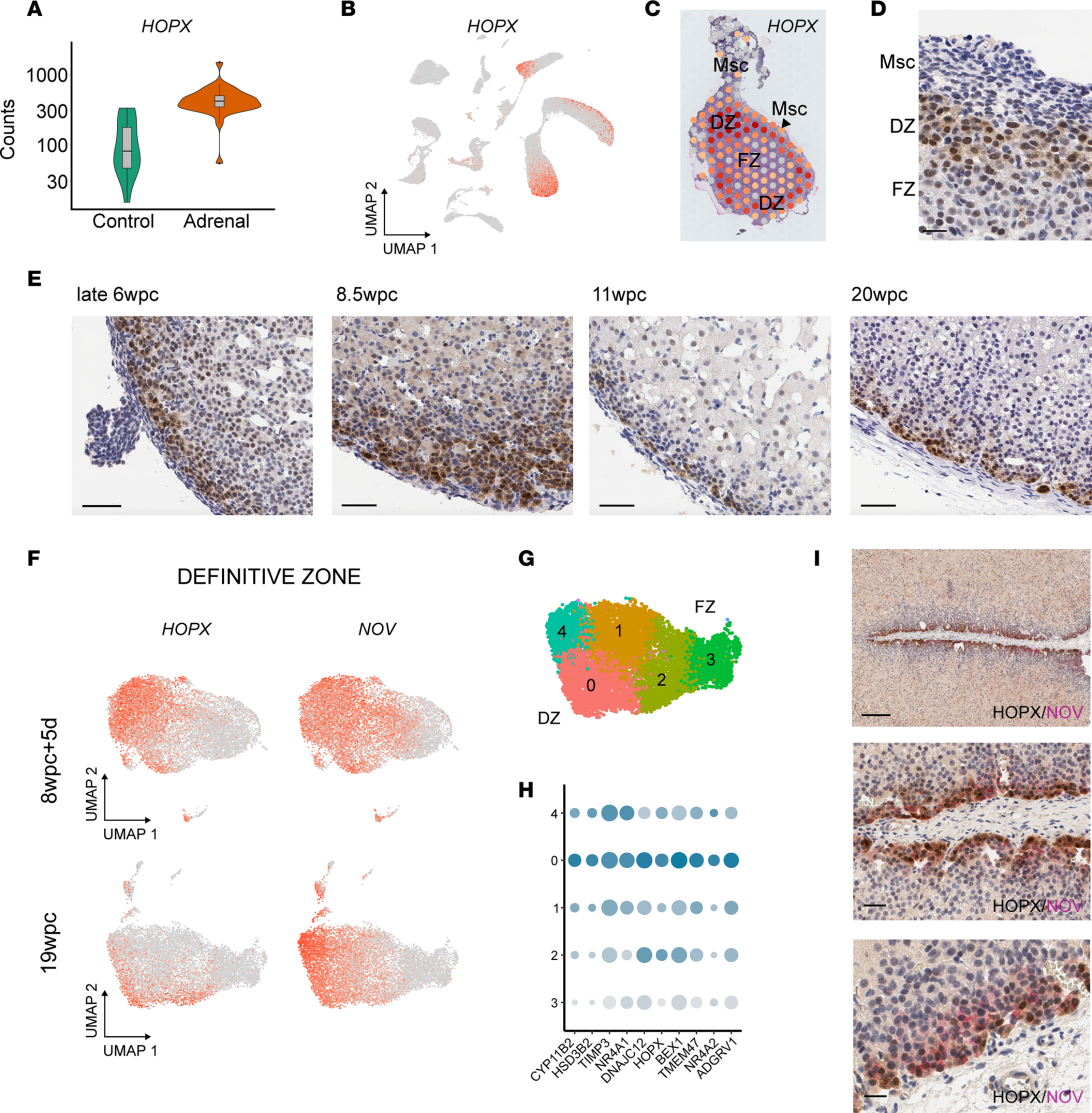
HOPX is a potentially novel DZ factor. (**A**) *HOPX* expression (normalized counts) in the human developing adrenal gland (combined adrenal gland samples, bulk RNA-Seq, *n* = 32) compared with controls (*n* = 14). (**B**) Feature plot of *HOPX* expression in the adrenal cortex clusters (for annotation, see Figure 1G). (**C**) Spatial transcriptomic spot plot showing DZ expression of *HOPX* at 7wpc+4d. Msc, mesenchyme. (**D**) Immunohistochemistry showing expression of *HOPX* in the DZ at late 6wpc between the layer of outer Msc and inner adrenal FZ. Scale: 20 μm. (**E**) Immunohistochemistry showing representative DZ expression of HOPX at each stage. Scales: all 50 μm. (**F**) Feature plots of *HOPX* expression in the adrenal cortex cluster at 2 different ages (8wpc+5d, 19wpc) compared with the DZ marker *NOV*. (**G**) UMAP of key cortex clusters at 19wpc. (**H**) Dot plot of the top differentially expressed genes in cluster 0 compared with other clusters at 19wpc. (**I**) Dual-labeled IHC of HOPX expression (brown) and NOV (magenta). Scales: 250 μm, *upper panel*; 50 μm, *center panel*; 20 μm, *lower panel*.

Although most of the “core” transcription factors identified showed expression throughout the adrenal cortex (i.e., DZ and FZ), an adrenally enriched gene that was expressed very strongly in the DZ compared with the FZ was *HOPX* (also known as Hop homeobox/homeobox-only protein) (Figure 5, A and B). HOPX is an atypical homeodomain protein that lacks direct DNA-binding capacity but interacts with transcriptional regulators to maintain quiescence in specific embryonic and adult stem cell populations and to control cell proliferation during organogenesis (50, 51). HOPX also acts as a tumor suppressor, and reduced HOPX expression is associated with several cancers (50, 51).

In our scRNA-Seq data set, *HOPX* was consistently one of the most differentially expressed markers of the DZ compared with the FZ in all ages studied (Supplemental Figure 15 and Supplemental Data 3). This strong enrichment of *HOPX* in the DZ was verified by spatial transcriptomic analysis, which showed a strong “ring” of *HOPX* DZ expression at 7wpc+4d (Figure 5C). This finding was validated by IHC, which showed that HOPX defined the outer border of the DZ at the interface of the peripheral mesenchyme at late 6wpc (Figure 5D). Furthermore, serial IHC analyses showed that HOPX was expressed in the outer DZ across time (late 6wpc–20wpc), marking the boundary between the developing adrenal gland and the mesenchyme (early) or subcapsular region of cells (later) (Figure 5E and Supplemental Figure 16).

As expected given its role in the DZ, *HOPX* colocalized in clusters with the DZ marker *NOV* in scRNA-Seq analysis, especially during early stages of development (Figure 5F). However, by 19wpc, *HOPX* expression was relatively reduced (Figure 4B and Figure 5F) and localized within a zG-like cluster that also expressed *HSD3B2*, *CYP11B2*, and the orphan nuclear receptors *NR4A1* (NURR77)/*NR4A2* (NUR1) (Figure 5, G and H, and Supplemental Figures 15 and 17). Of note, an emergent population of NOV-positive/HOPX-negative cells was identified by scRNA-Seq at 19wpc, which was located just central to the peripheral HOPX-positive cells on dual-labeled IHC (Figure 5I). Furthermore, *HOPX* does not seems to be strongly expressed in the mature adult human adrenal gland, consistent with the decreased expression of this gene seen with time (https://www.proteinatlas.org/ENSG00000171476-HOPX/tissue). Thus, HOPX likely plays a role in defining the human fetal adrenal DZ and emerging zG in early development and may maintain a specific population of cells in a replication state.

### Mesenchyme-cortex interactions during development.

**Figure 6:**
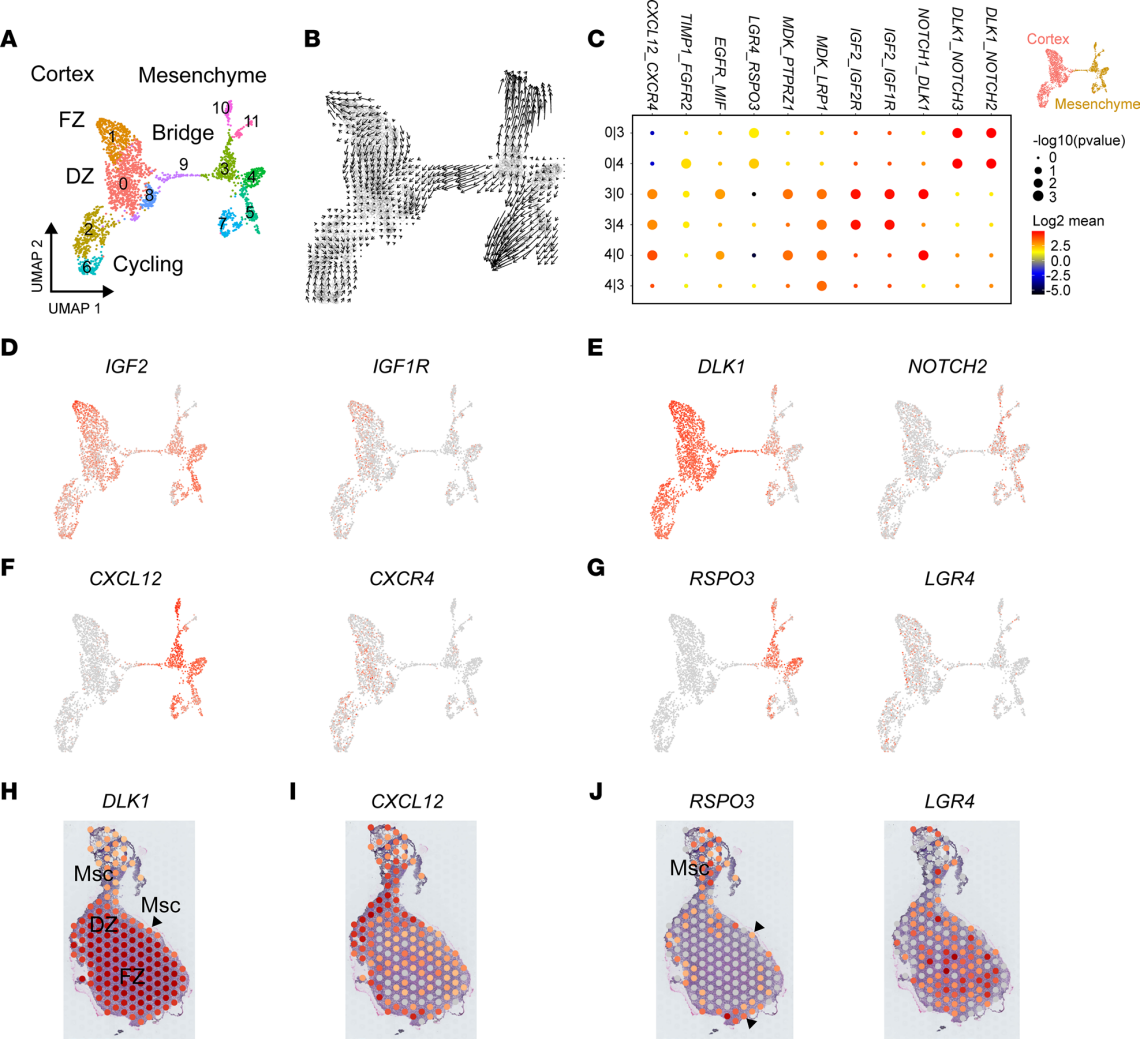
Potential bidirectional signaling interactions between the mesenchyme cluster and adrenal cortex. Data (scRNA-Seq) shown at 6wpc+6d. (**A**) UMAP of the mesenchyme and adrenal cortex clusters demonstrating the potential “bridge” between the 2 populations of cells. Subclusters used for cell-cell communication analysis are shown. (**B**) Single-cell velocity estimates overlaid on the UMAP of mesenchyme–adrenal cortex clusters (RNA velocity). (**C**) Potential ligand-receptor interactions for key subclusters in the mesenchyme (clusters 3, 4) and adrenal cortex (cluster 0), using CellPhoneDB. (**D**) Feature plot showing expression of *IGF2* (encoding ligand) and expression of *IGF1R* (encoding cognate receptor). (**E**) Feature plot showing expression of *DLK1* (encoding ligand) and expression of *Notch2* (encoding receptor) (see also Supplemental Figure 19). (**F**) Feature plot showing expression of *CXCL12* (encoding ligand) and expression of *CXCR4* (encoding receptor). (**G**) Feature plot showing expression of *RSPO3* (encoding ligand) and expression of *LGR4* (encoding receptor). (**H**) Spatial transcriptomic spot plot (7wpc+4d) of *DLK1* in the definitive DZ and FZ of the adrenal gland, with weaker expression in the mesenchyme (Msc)/subcapsular region. (**I**) Spatial transcriptomic spot plot (7wpc+4d) of *CXCL12*, strongest in the Msc/subcapsular region of the adrenal gland. (**J**) Spatial transcriptomic spot plot (7wpc+4d) of *RSPO3* in the Msc (arrowheads)/subcapsular region of the adrenal gland and *LGR4* in the adrenal cortex. *IGF2*, insulin-like growth factor 2; *RSPO3*, R-Spondin 3; *LGR4*, leucine-rich repeat-containing G protein–coupled receptor 4.

As the adrenal gland forms within a region of mesenchyme (Figure 5, C–E), more detailed analyses of potential ligand-receptor signaling interactions were undertaken using a combined cortex-mesenchyme scRNA-Seq data set. Notably, a potential transcriptomic “bridge” between the mesenchyme and cortex was identified in the merged adrenal data set, particularly in the 6wpc+5d sample (Figure 1G and Figure 6A). A trajectory of cells undergoing differentiation from the mesenchyme to cortex was also observed (Figure 6B). Several cluster-specific markers were identified (e.g., *LRRC3B*, *GRIA2*) (Supplemental Figure 18A and Supplemental Data 6). The nuclear receptors *NR2F1* (COUP-TF1) and *NR2F2* (COUP-TF2) showed a decreasing gradient of expression from the mesenchyme to adrenal, whereas *NR5A1* (SF-1) and *NR0B1* (DAX-1) expression increased (Supplemental Figure 18B).

Using CellPhoneDB (v2.0) (52) to investigate cell-cell communication networks and ligand-receptor interactions at this stage of adrenal gland development, several key systems were found to be enriched (Figure 6, C–G). For example, *IGF2* showed strong expression in mesenchyme and adrenal cortex, with strongest expression in the FZ, the region of highest expression of its cognate receptors, *IGF1R* and *IGF2R* (Figure 6, C and D, and Supplemental Figures 19 and 20). In contrast, *DLK1* (also known as PREF1) showed high adrenal cortex expression whereas the linked Notch family of receptors (which DLK1 represses) were expressed predominantly in the mesenchymal component (Figure 6, C, E, and H, and Supplemental Figure 19).

Two key signaling systems where ligands are potentially secreted from mesenchymal cells and have receptors in the developing adrenal cortex are *CXCL12* (encoding the ligand)/*CXCR4* (encoding the receptor) and *RSPO3* (ligand)/*LGR4* (receptor) (Figure 6, C, F, G, I, and J, and Supplemental Figure 19). *RSPO3*/*LGR4* are part of the WNT signaling system, and *RSPO3*/R-Spondin 3 has been proposed previously to be a key ligand released by subcapsular cells in both mouse and human adrenal development, with potential interactions with Lgr5 and zinc and ring finger 3 (Znrf3) (16, 53, 54). Using spatial transcriptomics, *RSPO3* expression was found to be expressed in the mesenchyme, including in an outer layer around the early adrenal gland (7wpc+4d), whereas *LGR4* was expressed more centrally in the FZ region (Figure 6, G and J). Strong *LGR5* and *LGR6* expression or interactions were not seen (Supplemental Figure 19). Thus, although several signaling systems have been proposed in adrenal development from data in the mouse (22, 23, 55), our unsupervised analysis of ligand-receptor interactions supports the roles of *IGF2*, *DLK1*, and *RSPO3*/R-Spondin 3 as major components in human adrenal development and suggest that *CXCL12* may also influence potential mesenchyme-adrenal interactions.

### Imprinted genes are enriched in the human fetal adrenal gland.

**Figure 7:**
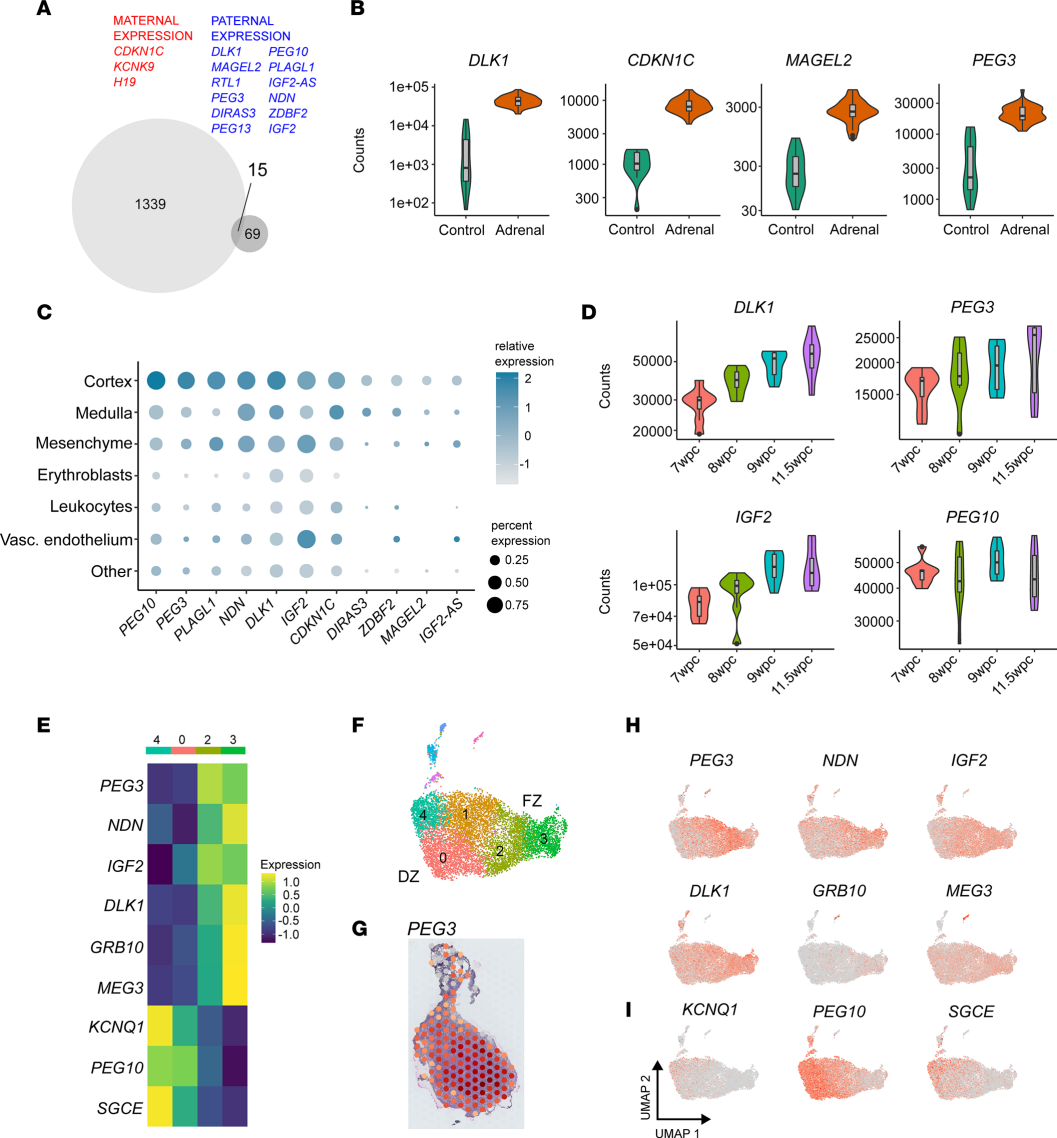
Imprinted genes in human adrenal development. (**A**) Venn diagram showing the 15 imprinted genes (non-placental-specific) that are differentially expressed in the adrenal cortex cluster (bulk RNA-Seq adrenal > control, log_2_FC > 1.5, padj < 0.05). (**B**) Violin plots (normalized counts) of bulk RNA-Seq expression of several key imprinted genes in the adrenal gland (*n* = 32) compared with control tissues (*n* = 14). (**C**) Dot plot of key differentially expressed imprinted genes in different scRNA-Seq clusters of the developing human adrenal gland. (**D**) Violin plots showing time series bulk RNA-Seq expression of key imprinted factors in the developing human adrenal gland (*n* = 8 at each stage). (**E**) Heatmap of the expression of key imprinted genes in different clusters of the adrenal cortex at 19wpc (see Figure 6F). (**F**) UMAP of adrenal cortex subclusters at 19wpc. (**G**) Spatial transcriptomic spot plot (7wpc+4d) of paternally expressed gene 3 (*PEG3*) showing strong expression, especially in the central FZ. (**H**) Feature plots of 6 key paternally expressed (maternally imprinted) genes in the adrenal cortex (19wpc). (**I**) Feature plot of 3 key maternally expressed (paternally imprinted) genes in the adrenal cortex (19wpc).

*IGF2* and *DLK1* are both imprinted genes, and it is well recognized that imprinted genes play a key role in many aspects of fetal and placental growth in humans (56). Paternally expressed (maternally imprinted) genes are frequently linked to growth promotion, whereas maternally expressed (paternally imprinted) genes are associated with growth restriction. To address the potential role of imprinted genes in the developing fetal adrenal gland in more detail, differential expression was initially studied using bulk RNA-Seq data (adrenal versus control, log_2_FC > 1.5, padj < 0.05). We found that 15 out of 84 (17.9%) well-established, non-placental-specific human imprinted genes (57) were differentially expressed in the adrenal gland, representing a significant enrichment of imprinted genes (15/1,354 versus 69/18,325, χ^2^ 15.9, *P* < 0.0001) (Figure 7, A and B, and Supplemental Data 7). Expression of these genes in adrenal cortex clusters was verified by scRNA-Seq analysis (Figure 7C). Several key paternally expressed genes were identified (e.g., *DLK1*, *PEG3*, *IGF2*, *PEG10*), often in the FZ (Figure 7, D–I). Taken together, these data highlight the important growth-promoting role paternally expressed genes, such as *IGF2* and *PEG3*, play in the human fetal adrenal cortex during early development, at a time of rapid adrenal gland growth (Figure 1B and Supplemental Figure 20).

### Adult adrenal transcriptomic expression and PAI.

**Figure 8:**
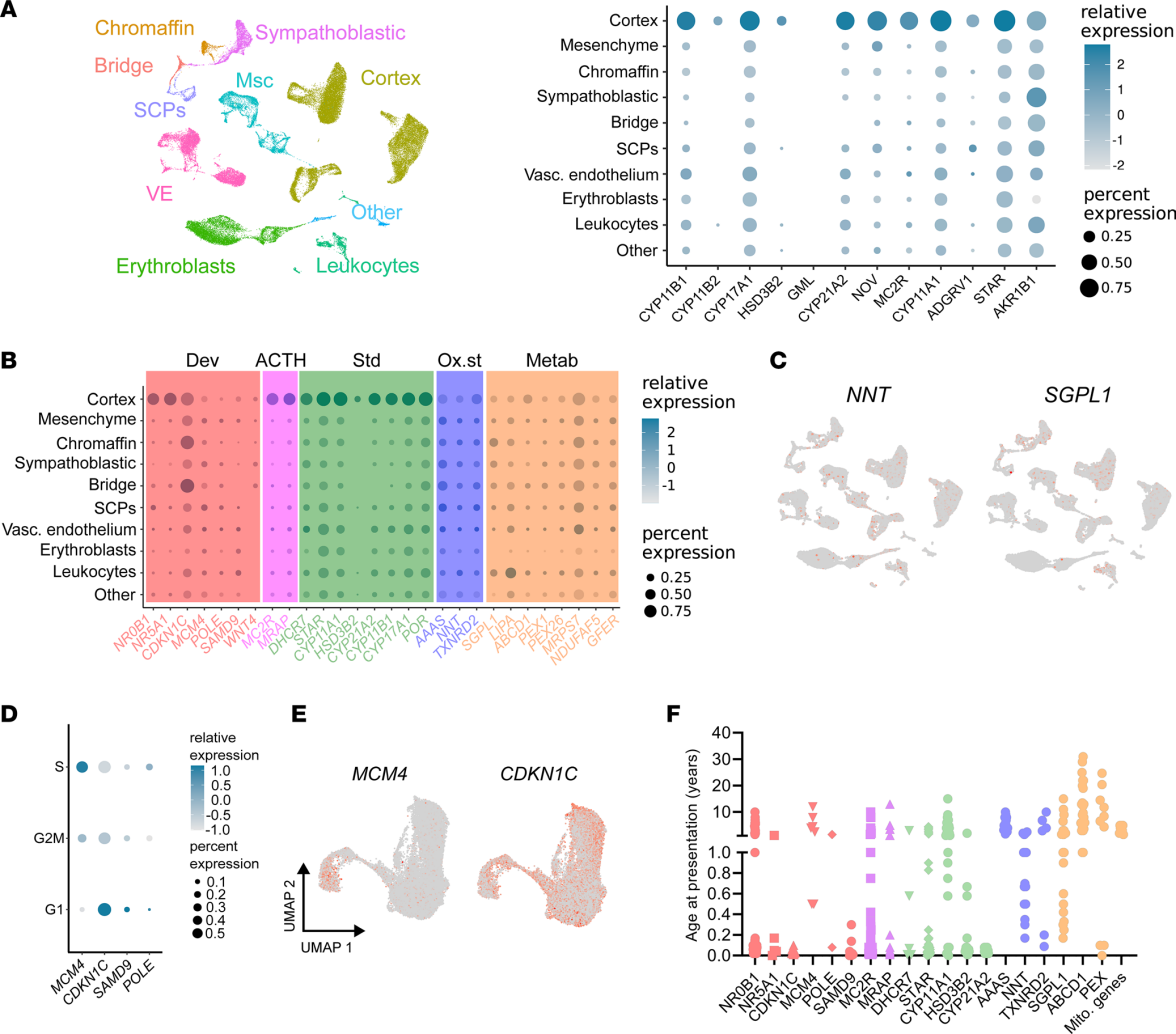
Expression of genes enriched in the adult adrenal gland and in monogenic causes of PAI. (**A**) Dot plot showing fetal adrenal gland expression of genes with the highest “tissue specificity score” (enriched expression) in the adult adrenal gland, as defined in the Human Protein Atlas (https://www.proteinatlas.org). VE, vascular endothelium; SCPs, Schwann cell precursors. (**B**) Dot plot showing the expression of genes associated with monogenic causes of primary adrenal (glucocorticoid) insufficiency (PAI) in the adrenal cortex and other adrenal clusters during development (see UMAP, **A**). Dev, developmental disorders; ACTH, ACTH resistance; Std, steroidogenic disorders; Ox. st, oxidative stress; Metab, metabolic disorders. (**C**) Feature plot for expression of nicotinamide nucleotide transhydrogenase (*NNT*) and sphingosine-1-phosphate lyase 1 (*SGPL1*). (**D**) Dot plot of the expression of PAI-causing genes proposed to be involved in adrenal growth and cell division in different cell cycle phases (S phase, G2M, G1). (**E**) Expression of mini-chromosome maintenance complex component 4 (*MCM4*) and cyclin-dependent kinase inhibitor 1C (*CDKN1C*) in the integrated adrenal cortex cluster with cycling cells included (see Figure 2I). (**F**) Age at presentation with adrenal insufficiency of children and young people with selected monogenic causes of PAI.

Finally, we considered how the transcriptomic profile of adrenal gland development relates to the adult adrenal gland, as well as to genes known to cause PAI. Using the top differentially expressed genes in the adult adrenal gland (*n* = 12) (Human Protein Atlas, https://www.proteinatlas.org/humanproteome/tissue/adrenal+gland), we found consistent correlations with many differentially expressed genes during development (Figure 8A). However, the gene encoding glycosylphosphatidylinositol anchored molecule like (*GML*) was not present in the fetal data, and several other genes were predominantly expressed in later fetal adrenal stage (19wpc) (e.g., *HSD3B2*, *CYP11B2*, *ADGRV1*) (Supplemental Figure 21). This finding contrasts with *HOPX*, which is predominantly expressed in the fetal adrenal but not in the adult organ.

We also analyzed developmental expression of genes known to be monogenic causes of PAI (Figure 8B) (10). Most key transcription factors (e.g., *NR5A1*, *NR0B1*), components of steroidogenesis (e.g., *STAR*, *CYP11A1*, *CYP21A2*) and genes involved in ACTH signaling (e.g., *MC2R*, *MRAP*) showed high specificity for expression in the fetal adrenal cortex cluster (Figure 8B). However, many genes linked to oxidative stress processes or metabolic function were expressed at low levels in multiple clusters (e.g., *NNT*, *AAAS*, *SGPL1*, *ABCD1*) (10) (Figure 8, B and C). In addition, out of those genes associated with multisystem growth restriction phenotypes (e.g., *MCM4*, *CDKN1C*, *SAMD9*, *POLE*) (58–60), only *MCM4* (associated with PAI, short stature, natural killer cell deficiency) (58) was expressed predominantly in cycling cells (S phase) (Figure 8, B, D, and E). Of note, when the age of presentation of children with classic monogenic causes of PAI was analyzed, it emerged that children who had disruption of highly adrenal cortex/adrenal–specific genes often presented with adrenal insufficiency soon after birth (in the first 2 weeks), whereas those children with defects in genes with less adrenal cortex–specific profiles often had a delayed clinical presentation in later infancy, childhood, or even adult life (χ^2^ 7.46, *P* < 0.006) (Figure 8F; for details of analysis and data, see Supplemental Data 8).

## Discussion

This study provides one of the first detailed investigations into the complexities of human adrenal gland development up to 20wpc and demonstrates the benefits of integrating transcriptomic data (bulk RNA-Seq, scRNA-Seq, spatial) with developmental anatomy and physiology when investigating the biological basis of organogenesis and related clinical conditions.

It is already established that the human adrenal gland undergoes marked growth throughout gestation and at birth is approximately the same weight as in adult life (33, 34). Much of this growth is due to the expansion of the large FZ, which is found only in humans and higher primates. Here, we document changes in growth and morphology up to 20wpc. Using scRNA-Seq analysis of cycling cells, coupled with IHC markers of cell division (Ki-67), we show that there is rapid cell division during the late embryonic/early fetal stage and that more dividing cells are located in the outer DZ region. A potential trajectory of cell differentiation from the DZ to FZ was seen during early adrenal development (22, 23, 43, 61, 62). Imprinted genes, such as *IGF2* and *DLK1*, play a key role in adrenal growth (32, 56, 63, 64). Here, we demonstrate strong expression of paternally expressed growth-promoting genes, especially in the FZ region, consistent with the rapid growth seen during this stage of development.

Other key findings were the marked increase in vascularization of the adrenal gland across this time frame and development of vascular sinusoids within the FZ. Recently developed imaging techniques, such as micro-CT (65), highlighted the surface arrangement of these vascular networks, especially on the inferior aspect of the adrenal gland that is adjacent on the upper pole of the kidney. Studies of angiogenesis and vascular remodeling in the fetal adrenal gland have focused on both the VEGF/VEGFR1 and angiopoietin/Tie systems (66–68). Here, we demonstrate the key role for *FLT1* (encoding VEGFR1). Vascular channels are crucial for transporting large numbers of adrenal androgens into the fetal circulation, with subsequent placental conversation to estrogens, and may influence cortico-medullary interactions. The late embryonic and fetal period is a key time when these vascular systems are established.

Although the main role of the adult adrenal cortex is the biosynthesis and release of steroid hormones (mineralocorticoids, glucocorticoids, androgens), the extent to which these hormones can be generated in the fetal adrenal gland remains to be fully elucidated. Recent studies have looked at expression of key components of these pathways or attempted to measure the major steroid hormones and their metabolites directly (34, 35, 39). Here, we show that the FZ has the transcriptomic machinery to secrete large numbers of adrenal androgens, such as DHEA(-S). Precursors are shunted into this pathway because of the lack of *HSD3B2*. Expression of the ACTH receptor (*MC2R*) and its accessory protein (*MRAP*) increased with age and showed strong expression in the FZ region. This finding is in keeping with ACTH-dependent stimulation of androgens in fetal adrenal cell or tissue cultures, suggesting the FZ androgen biosynthesis may have the capacity to be ACTH driven (69, 70).

In contrast, glucocorticoid biosynthesis (e.g., cortisol) requires *HSD3B2* expression. Consistent with 3 previous reports (33, 35, 39), we detected a potential transient increase in *HSD3B2* at around 8.5wpc. This pattern was similar in 46,XY and 46,XX samples, rather than just a 46,XX phenomenon as recently suggested (39). However, *HSD3B2* expression was stronger and more consistent by 19wpc in DZ cells that often coexpressed *CYP21A2* and *CYP11B1*, both of which increased with time. Very limited expression of the genes required for mineralocorticoid biosynthesis (e.g., aldosterone) was seen early on, but a small proportion of DZ cells did express *CYP11B2* with other relevant enzymes by 19wpc. This finding is consistent with a lack of aldosterone synthesis in the first half of gestation, although increases in *CYP11B2* expression toward the end of the second trimester suggest the capacity for aldosterone synthesis is being established (34, 35). Of note, preterm babies often have hypotension and salt loss, which may in part be due to immature development of mineralocorticoid biosynthesis, as well as relative mineralocorticoid resistance. Understanding the dynamic transcriptomic and physiological changes around this time is key.

Two key transcription factors (TFs) that regulate fetal adrenal development are the nuclear receptors *NR0B1* (DAX-1) and *NR5A1* (SF-1) (14, 17, 19, 20, 71). These genes encode 2 important nuclear receptors within a “core” set of 17 TFs identified, which were consistently differentially expressed in the adrenal cortex across time. Another transcription regulator identified that was remarkable for its consistent differential expression in the DZ compared with the FZ was *HOPX* (50). HOPX is an atypical homeobox factor that lacks direct DNA binding and likely interacts with transcriptional regulators (50), so it is not universally classified as a TF. Nevertheless, HOPX is emerging as a key embryonic and adult stem cell marker involved in stem cell maintenance/quiescence (72, 73) and controlled tissue differentiation (50). HOPX is emerging as having a role in the development of mesoderm progenitor cells/hematopoietic stem cells (73, 74), osteogenic cells, neuronal tissue (72), cardiomyoblasts (75), intestinal crypt/colonic cells (51, 76), skin (77), alveolar epithelial cells (type I) (78), and endometrium. HOPX can influence tissue repair and regeneration (76), and reduced *HOPX* expression (through promoter methylation) is associated with several cancers — e.g., colon (51), breast (79), thyroid, pancreas (80) — and metastasis risk — e.g., nasopharyngeal (81) — suggesting HOPX acts as a tumor suppressor. Interactions with WNT signaling (75), activated SMAD (75), and CXCL12 (73) have also been proposed.

Differential expression of HOPX in the human adrenal FZ has recently been reported by Cheng et al. (16), who suggested that it may play a role in maintaining stemness in these cells. However, data were only shown to 8wpc and in a limited number of cells. Here, we expand our insight into HOPX in the developing human adrenal gland into later gestation and at the protein level. We identified HOPX as a “core” transcription regulator and showed clear differential expression in the adrenal gland compared with other tissues (bulk RNA-Seq). Using spatial transcriptomics and IHC, we demonstrated that both the *HOPX* gene and the protein were clearly expressed at the outer boundary of the developing DZ, close to the mesenchymal layer initially and in the subcapsular part of the DZ later. A potential decrease in HOPX was seen with age, consistent with low expression of *HOPX* in adult adrenal databases. Furthermore, using scRNA-Seq and dual-labeled IHC, we showed colocalization of HOPX with NOV but also the presence of a HOPX-negative/NOV-positive population of cells at 19wpc. Given the decrease of HOPX with age, it is possible that HOPX plays a role in maintaining controlled cell proliferation and growth in the developing DZ. Of note, Xing et al. showed in 2010 that *HOPX* is downregulated following ACTH stimulation in studies of human adult and fetal adrenal cells in vitro, whereas ACTH stimulates synthesis of steroidogenic enzymes (69). Coupled with our observation of strongest *MC2R*/*MRAP* expression in the FZ, we hypothesize that ACTH and its pathway promote adrenal differentiation not only by upregulating steroidogenesis but also by downregulating *HOPX*/HOPX and allowing cells to actively differentiate. Certainly, the role for HOPX in the DZ during early development and differentiation needs further investigation.

As the adrenal gland arises from a condensation of intermediate mesoderm/mesenchyme at around 4wpc, we also focused on mesenchyme-cortex interactions during the earliest phase of development investigated (6wpc to 8wpc). Indeed, IHC and spatial transcriptomic analysis clearly showed the adrenal gland developing within an outer ring of mesenchymal cells next to a mesenchymal “pedicle.” The early adrenal gland had a bulk transcriptomic profile closer to the kidney (mesoderm) initially that became increasingly distinct with time, as the relative proportion of mesenchymal cells diminished and that of adrenal specific cells increased. Studying mesenchyme-cortex clusters at 6wpc, we identified a potential trajectory of cells differentiating from the mesenchyme to the cortex, consistent with a pool of progenitor cells in the region, which ultimately located within the subcapsular region (24, 82, 83). Several signaling systems have been proposed to regulate mesenchyme-cortex interactions from studies in the mouse (22–24, 84, 85). Using an unsupervised approach of CellPhoneDB (52) to identify ligand-receptor interactions, we found that R-Spondin 3 (*RSPO3*) could have an important role. R-Spondin 3 is a component of the WNT signaling pathway; has been shown to be expressed in the subcapsular region of cells in the developing mouse adrenal gland (53), as well as in subcapsular cells in the 8wpc human adrenal gland (16); and potentially mediates a gradient of WNT signaling involved in adrenal zonation. Although interactions with LGR5 have been suggested (53), we identified LGR4 as the most likely expressed putative cortex receptor. Of note, a recent report of an iPSC model of human fetal adrenal development showed the emergence of an *RSPO3*-expressing cluster with capsule cell–like properties, as well as potential regulation of *LGR4* by NR5A1/SF-1 (40). These findings support our conclusion that R-Spondin 3/LGR4 interactions may be key. Furthermore, a potential role for *CXCL12* (mesenchymal ligand) and *CXCR4* (adrenal cortex receptor) was also identified. Other signaling systems proposed from mouse models (e.g., *Shh*/*Gli*) were not found to be strongly expressed in the developing human adrenal gland at this stage. Taken together, these data suggest R-Spondin 3–driven WNT signaling has a key role in human adrenal development, as well as in mice.

Our findings also address how basic biological mechanisms relate to human disease. Our translational focus over the years has been on monogenic causes of PAI. In children and young people these conditions are often inherited and represent potentially life-threatening disorders needing prompt diagnosis and management (9). Progress over the past 3 decades has identified around 30 single-gene causes of PAI (10), some of which are shown here to have specific developmental features — e.g., *NR5A1*/*NR0B1* as core transcriptional regulators (20) and *MCM4* in S phase cell division (58). The differentially expressed adrenal genes our analyses found will provide candidate genes for new genetic causes of PAI. Several key genes associated with PAI were not strongly differentially expressed in the developing fetal adrenal cortex (e.g., *NNT*, *SGPL1*, *AAAS*, *ABCD1*). These genes have been proposed to regulate oxidative stress or metabolic processes. Of note, conditions linked to these genes rarely present with PAI in the first 3 months of life. We hypothesize therefore that, for these conditions, a period of postnatal stress may be required that leads to gradual decompensation and then clinical adrenal insufficiency. Making an early diagnosis — potentially even through newborn genetic screening programs — means a window of opportunity exists to alter the disease course, or at least to predict the onset of PAI and avoid an adrenal crisis.

Insights into basic mechanisms of human adrenal development also have implications for better understanding the drivers of adrenal tumors. We have previously shown the opposing effects of variants in *CDKN1C*/CDKN1C, whereby gain of function of this cell cycle repressor is associated with adrenal hypoplasia and IMAGe syndrome and loss of function is associated with Beckwith-Wiedemann syndrome with an adrenal neoplasm risk (59). In childhood especially, adrenocortical tumorigenesis has been linked to increased expression of *NR5A1* (86, 87) and *IGF2* (through aberrant regulation of the 11p H19/IGF2 imprinting locus) (88–90), and IGF1R blockade has been explored as a treatment for adrenal tumors in experimental models and trials (91–93). Thus, the association of imprinted genes (e.g., *CDKN1C*, *IGF2*) with growth and tumor risk is emerging. More recently, CXCR4 expression has been used as a marker and potential therapeutic target in adrenal cancer (94), as well as for clinical diagnostic imaging of aldosterone-secreting adenomas using ^68^Ga-pentixafor PET/CT (95). Our findings also have relevance for the mechanisms of adrenal androgen synthesis and regulation in CAH (e.g., 21-hydroxylase deficiency) (3, 36, 44), for insights into adrenarche and links between the FZ and zona reticularis (5, 96, 97), and for potential “programming” of the hypothalamic-pituitary-adrenal (HPA) axis during development, which could have implications for postnatal variability in HPA axis function and stress responses (98).

These data have several limitations. The developmental period of tissue accessibility was somewhat limited, and a greater sample number over time would have provided more detailed data. Bulk RNA-Seq reflects both cell number and transcript expression per cell, whereas scRNA-Seq may have limited sensitivity at low expression levels. While scRNA-Seq and spatial transcriptomic platforms provide significant new insight, the ability to obtain increased sequencing reads per cell, more cells sequenced per sample, or greater spatial resolution is always improving and will help address some of the hypotheses generated here in the future. Understanding anatomical and physiological relations during development will be key going forward, at gene transcription, RNA expression, and protein levels, and integrating detailed histology and imaging with basic cell biology will be crucial, as we have attempted to do here.

In summary, this study highlights the unique developmental complexities of human fetal adrenal gland development up to midgestation and provides an integrated transcriptomic roadmap with potential long-term consequences for human health and disease.

## Methods

### Tissue samples.

Human embryonic and fetal tissue samples used for bulk RNA Seq, IHC, and micro-CT were obtained with ethical approval (REC references: 08/H0712/34+5, 18/LO/0822, 08/H0906/21+5, 18/NE/0290) and informed consent from the joint Medical Research Council (MRC)/Wellcome Trust–funded Human Developmental Biology Resource (HDBR) (http://www.hdbr.org). HDBR is regulated by the UK Human Tissue Authority (HTA; www.hta.gov.uk) and operates in accordance with the relevant HTA Codes of Practice. The age of embryos up to 8wpc was calculated based on Carnegie staging, whereas in older fetuses the age was estimated from foot length and knee-heel length in relation to standard growth data. Samples were karyotyped by G-banding or quantitative PCR (chromosomes 13, 16, 18, 21, 22, and X and Y) to determine the sex of the embryo/fetus and to exclude any major chromosomal rearrangements or aneuploidies. The acquisition of adrenal samples used to generate scRNA-Seq data and spatial transcriptomics has been described previously (21), under the following studies: NHS National Research Ethics Service reference 96/085 (fetal tissues) and the joint MRC/Wellcome Trust–funded HDBR (as above). An overview of all samples used in the study is provided in Supplemental Data 1. Samples were stored in the appropriate media or at –80°C until processing. Adrenal dimensions were measured to the nearest 0.5 mm, using a light microscope (ZEISS Stemi 2000-C) when necessary. Adrenal weights (single gland, 10% formalin) were measured on an analytical balance (Pioneer PX) after removal of surface liquid.

### Micro-CT.

The 17wpc adrenal gland studied (10% formalin) was immersed in 1.25% potassium tri-iodide at room temperature for 48 hours, then rinsed, dried, and-wax embedded (99). Once hardened, excess wax was trimmed in order to preserve tissue shape, to reduce dehydration and movement artifact, and to optimize contact with the x-ray beam source. Micro-CT scans were carried out using a Nikon XTH225 ST scanner (Nikon Metrology) with the following settings: Tungsten target, x-ray energy 110 kV, current 60 μA (power 6.6 W), exposure time 1,420 ms, 1 frame per 3,141 projections, detector gain 24 dB, and scan duration of 75 minutes. Modified Feldkamp filtered back projection algorithms were used for reconstructions within proprietary software (CTPro3D; Nikon Metrology) and postprocessed using VG StudioMAX (Volume Graphics) to create the images at 4.77 μm isotropic voxel sizes.

### Bulk RNA-Seq.

Total RNA was extracted from human fetal adrenal samples (*n* = 32; Figure 1A and Supplemental Figure 1) and controls (*n* = 14, Figure 1A and Supplemental Figure 1, balanced across the age range) using the AllPrep DNA/RNA Mini Kit (QIAGEN) (RNA integrity number > 7.0). cDNA libraries were prepared using the KAPA mRNA HyperPrep Kit (Roche) and subsequently sequenced on a NextSeq 500 sequencer (paired-end 43 bp) (Illumina) in a single run to reduce potential batch effects. Fastq files were processed by FastQC and aligned to the human genome (Ensembl, GRCh 38.86) using STAR (2.5.2a). The matrix containing uniquely mapped read counts was generated using featureCounts, part of the R package Rsubread. MultiQC was used to visualize results (Supplemental Figure 22). Differential expression analysis was performed using DESeq2, using 8 control samples instead of 14 where indicated to prevent duplication of specific tissue types. Heatmaps for distances between samples and differentially expressed genes in adrenal versus control samples were generated using the pheatmap library in R.

### scRNA-Seq.

Detailed methods have been reported previously for the single-cell sequencing of the samples used, including fetal adrenal single-cell dissociation, Chromium processing (Chromium Single Cell 3′ kit) (10X Genomics), cDNA library preparation and sequencing (Illumina HiSeq 4000), together with quality control analysis (21). A processed single-cell matrix was generated as described before (21) with minor modifications. Unless specified, cycling cells were discarded from the analysis. The R package Seurat (v4.0.2) (100) was used for processing the single-cell matrix. Briefly, the count matrix was normalized and 2,000 highly variable genes were chosen. After gene scaling, dimensionality reduction was performed using the first 75 principal components. The FindClusters and RunUMAP functions were used to identify clusters and to allow UMAP visualization. The clustree package in R was used to select the resolution parameter for clustering. Details regarding cluster annotation markers and algorithmically defined markers are described in Kildisiute et al. (Methods and Supplemental Tables 2 and 5 in ref. 21). Differentially expressed genes between clusters were calculated using the FindAllMarkers or FindMarkers functions using the parameters min.pct=0.25 and logfc.threshold=0.25 (Wilcoxon rank-sum test). Internal functions in Seurat (FeaturePlot, RidgePlot) were used to visualize marker expression. The FeatureScatter function was used to generate plots for pair of genes. The dittoSeq Bioconductor package was used to generate bar plots, heatmaps, and dot plots. RNA velocity on selected fetal adrenal samples was calculated using velocyto and plotted using the velocyto.R package in R as described before (21). Adrenal cortex sample integration was performed using data sets normalized with SCT as described in vignettes (Seurat). Cell-cell communication by ligand-receptor interactions was calculated using CellPhoneDB v.2.0 (52).

### Histology/IHC.

Human embryonic/fetal adrenal glands at 4 ages (“late 6wpc,” 8.5wpc, 11wpc, 20wpc) were fixed in 4% paraformaldehyde before being processed, embedded, and sectioned for histological analysis and IHC. Standard H&E staining was performed on 4 μm sections to show key structural regions and vasculature. IHC was undertaken on 4 μm sections using a BOND-MAX automated platform (Leica Biosystems). In brief, sections first underwent antigen retrieval to unmask the epitope (heat induced epitope retrieval [HIER], BOND-MAX protocol F), endogenous activity was blocked with peroxidase using a BOND polymer refine kit (catalog DS9800), and then incubation was undertaken with the relevant primary antibody for 1 hour. The following primary antibodies were used: VEGFR1 (Thermo Fisher Scientific PA1-21731, 1:100 dilution, HIER1 for 20 minutes), KI67 (Leica ready-to-use clone K2 PA0230, 1:100, HIER2 for 20 minutes), NOV (CCN3) (MilliporeSigma HPA019864, 1:100, HIER1 for 20 minutes), SULT2A1 (MilliporeSigma HPA041487, 1:100, HIER2 for 20 minutes), and HOPX (MilliporeSigma HPA030180, 1:100, HIER2 for 20 minutes). Next, the post-primary antibody was applied to the sections (BOND polymer refine kit) and HRP-labeled polymer, followed by DAB chromogen solution to precipitate the locus of antigen-antibody interactions (all BOND polymer refine kit). Sections were counterstained with hematoxylin, washed in deionized water, dehydrated in graded alcohols, cleared in 2 xylene changes, and mounted for light microscopy. The stained slides were imaged on an Aperio CS2 Scanner (Leica Biosystems) at 40× objective. Analysis was undertaken with QuPath (v.0.2.3) (https://qupath.github.io) and ImageScope (Leica Biosystems) software.

Dual staining was performed with anti-HOPX (1:100 dilution) and anti-NOV (1:100 dilution) antibodies (as above) on 20wpc human fetal adrenal gland. Antigen retrieval was heat induced, HIER2 20 minutes. Staining was performed sequentially on the BOND-MAX autostainer using anti-HOPX detected by brown chromogen (BOND polymer refine kit, catalog DS9800) and anti-NOV detected by red chromogen (BOND polymer red kit, catalog DS9390).

### Spatial transcriptomic analysis.

Spatial transcriptomic analysis of a single adrenal gland (7wpc+5d) was undertaken based on a standard 10X Genomics Visium protocol. In brief, the fresh adrenal sample was snap-frozen and embedded in OCT. Cryosections (10 μm) were cut and placed on Visium slides. Sections were fixed in cold methanol and stained with H&E to visualize the structures and tissue integrity, before permeabilization, reverse transcription, and cDNA synthesis. Second-strand cDNA was liberated and libraries (single-index) were generated by a PCR-based protocol. Libraries were sequenced on a HiSeq400 sequencer (Illumina). Sequencing data were aligned to GRCh38 human reference genome using Space Ranger Software to quantify gene counts in spots.

### Adult adrenal gland gene enrichment.

Data for the most highly differentially expressed (enriched) adult adrenal gland genes were derived from the Human Protein Atlas, using the “tissue specificity score” (TS). The TS represents the fold-change between the expression level in adrenal gland and that in the tissue with the second-highest expression level (https://www.proteinatlas.org/humanproteome/tissue/adrenal+gland).

### PAI clinical presentation.

Data for the clinical age of presentation of children and young people with genetic causes of PAI were obtained from original case series reports of classic and nonclassic conditions using PubMed (https://pubmed.ncbi.nlm.nih.gov/; accessed July 2022). Relevant literature sources are shown in Supplemental Data 8. Where PAI is a rare association of a condition, or where limited data are currently available, all published individual case reports were reviewed by 2 observers. Early-onset PAI was defined as having at least 1 report of an infant presenting with adrenal insufficiency within the first 2 weeks of life and late-onset PAI after this time. PAI-associated genes were termed adrenal specific if bulk RNA-Seq data showed greater expression of that gene in the adrenal gland compared with controls (log_2_FC > 2, padj < 0.05) and if expression in the integrated adrenal cortex cluster (or cortex and chromaffin) was high (Supplemental Data 8).

### Statistics.

Statistical analysis for bulk- and single-cell RNA-Seq data is described above within packages of differential expression analysis, with Bonferroni’s adjustments for multiple corrections. GraphPad Prism was used for χ^2^ analysis. In all analyses, a *P* value or adjusted *P* value less than 0.05 was taken as significant.

### Study approval.

Human tissue was obtained with written, informed consent under the following study approvals: 08/H0712/34+5, 18/LO/0822 (both London, United Kingdom — Fulham Research Ethics Committee), 08/H0906/21+5, and 18/NE/0290, 96/085 (both Newcastle, United Kingdom — North East Newcastle & North Tyneside 1 Research Ethics Committee).

### Data availability.

Single-cell RNA-sequencing data are deposited in the European Genome-phenome Archive (accession code EGAD00001008345). Bulk RNA-sequencing data are deposited in ArrayExpress/Biostudies (accession number E-MTAB-12492).

Values for all data points found in graphs are in the Supporting Data Values file.

## Author contributions

Study conceptualization was performed by IDV, SB, and JCA; methodology was developed by IDV, MDY, and ICS; investigation was performed by IDV, MDY, OKO, ICS, FB, BC, NM, TB, PN, KS, JPS, SMMB, and JCA; formal analysis was performed by IDV, MDY, GK, ICS, EK, and FB; data curation was performed by IDV, MDY, and FB; project administration was performed by JCA; supervision was provided by OJA, SB, and JCA; validation was performed by IDV, MDY, and JCA; visualization was performed by IDV, OKO, ICS, FB, and JCA; writing of the original draft was performed by IDV and JCA; review and editing were performed by all authors; and funding was acquired by SB and JCA.

## Supplementary Material

Supplemental data

Supplemental data sets 1-8

Supplemental video 1

Supporting data values

## Version 2 07/24/2023

Electronic publication

## Version 1 06/06/2023

Electronic publication
