# Supplementary material for: An integrated single-cell analysis of human adrenal cortex development
Source: JCI Insight. 2023 Jul 24;8(14):e168177. doi: 10.1172/jci.insight.168177 (PMC10443814; doi:10.1172/jci.insight.168177)
Supplement: Supplemental data [file jciinsight-8-168177-s114.pdf]

Supplemental Figures

An integrated single cell atlas of human adrenal cortex development

Ignacio del Valle[1], Matthew D Young[2], Gerda Kildisiute[2], Olumide K Ogunbiyi[3,4], Federica Buonocore[1], Ian C Simcock[5,6], Eleonora Khabirova[2], Berta Crespo[4], Nadjeda Moreno[4], Tony Brooks[7], Paola Niola[7], Katherine Swarbrick[3,4], Jenifer P Suntharalingham[1], Sinead M McGlacken-Byrne[1], Owen J Arthurs[5], Sam Behjati[2,8,9†], John C Achermann[1†*]

[1]Genetics and Genomic Medicine Research and Teaching Department, UCL Great Ormond Street Institute of Child Health, University College London, London, WC1N 1EH, UK; [2]Wellcome Sanger Institute, Wellcome Genome Campus, Hinxton, Cambridge, CB10 1SA, UK; [3]Department of Histopathology, Great Ormond Street Hospital for Children NHS Foundation Trust, London, WC1N 3JH, UK; [4]Developmental Biology and Cancer Research and Teaching Department, UCL Great Ormond Street Institute of Child Health, University College London, London, WC1N 1EH, UK; [5]Department of Clinical Radiology, Great Ormond Street Hospital for Children NHS Foundation Trust, London, WC1N 3JH, UK; [6]Population, Policy and Practice Research and Teaching Department, UCL Great Ormond Street Institute of Child Health, University College London, London, WC1N 1EH, UK; [7]UCL Genomics, Zayed Centre for Research, UCL Great Ormond Street Institute of Child Health, University College London, London, WC1N 1DZ, United Kingdom; [8]Cambridge University Hospitals NHS Foundation Trust, Cambridge, CB2 0QQ, UK; [9]Department of Paediatrics, University of Cambridge, Cambridge, CB2 0QQ, UK.

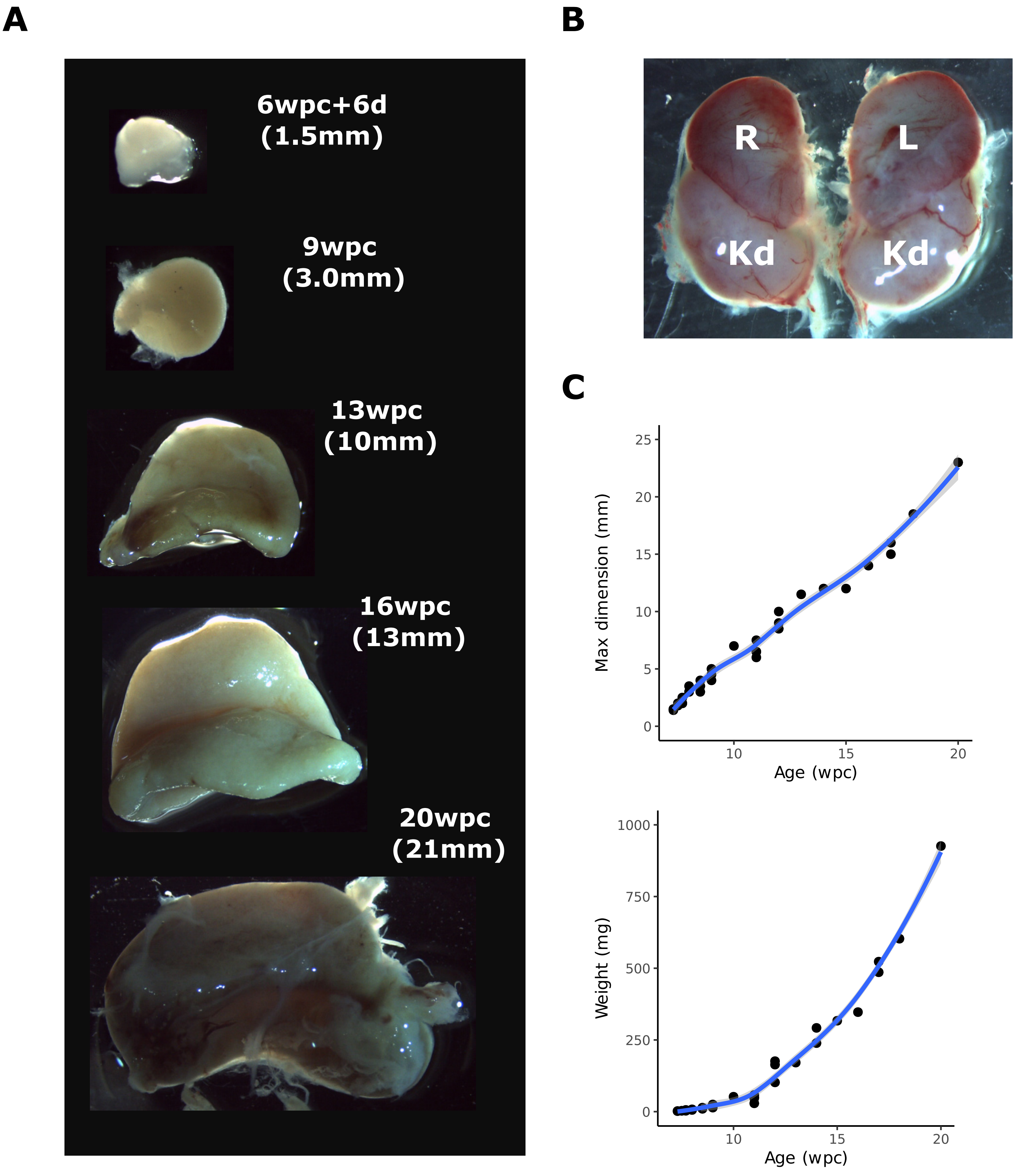

Supplementary Figure 1. Overview of human adrenal growth. A Photographs of single adrenal glands at 6 weeks post conception (wpc)+6 days (d), 9wpc, 13wpc, 16wpc and 20wpc (not to scale). The corresponding maximum dimension is indicated. **B** Photograph of the right (R) and left (L) adrenal glands above the kidneys (Kd) at 14wpc. **c** Growth plots for adrenal size (maximum dimension) (*upper panel*) and weight (*lower panel*) between 7wpc and 20wpc (n=36).

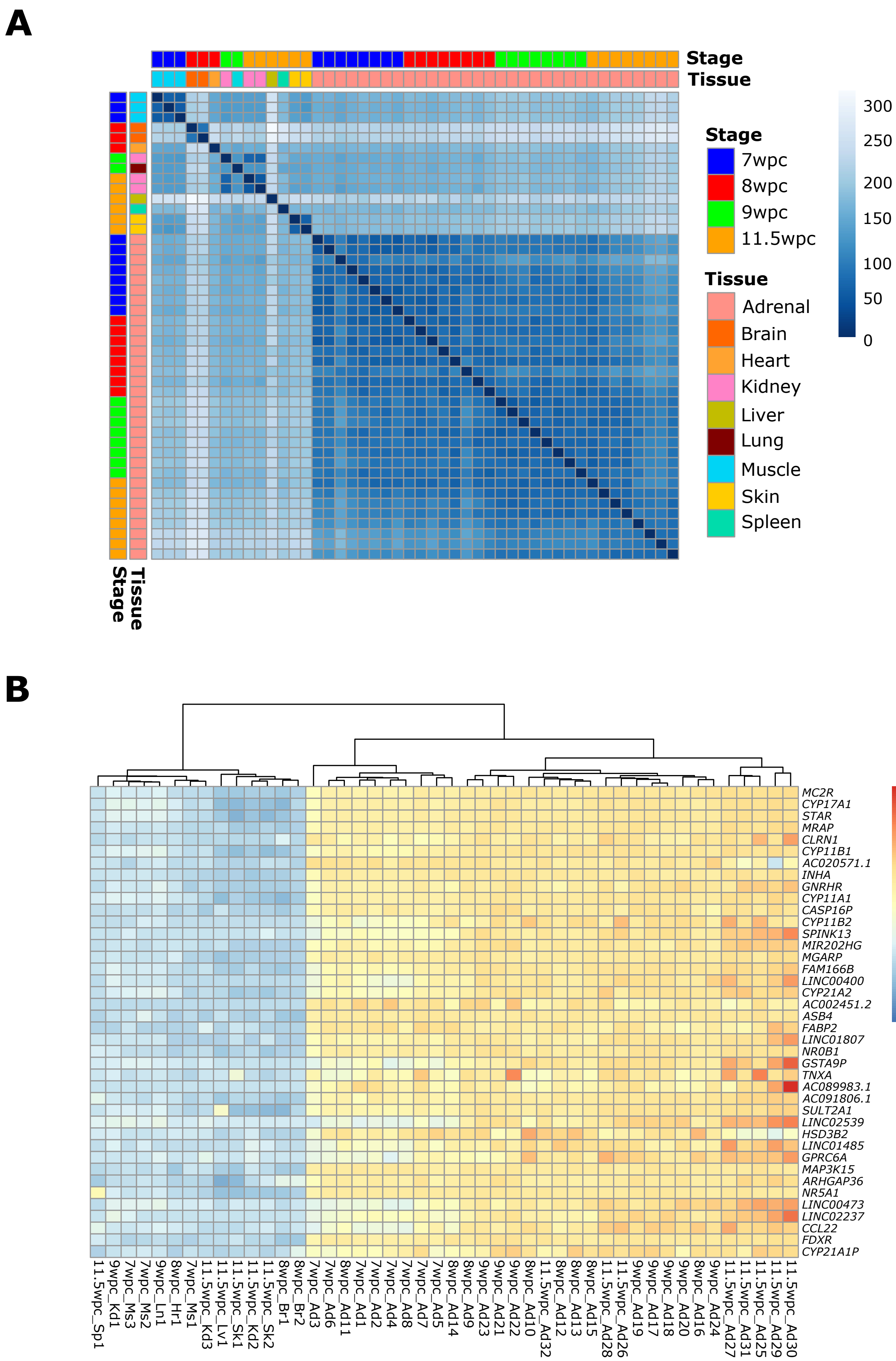

Supplemental Figure 2. Gene expression heatmaps across the bulk RNA-seq datasets. A Correlation plot of gene expression in adrenal glands (n=32) and controls (n=14) across the time series. The origin and age of each tissue sample is shown. **B** Unsupervised clustering heatmap to show the top 50 differentially-expressed genes (based on fold change) in the adrenal samples compared to control tissues. Ad, adrenal; Br, brain; Hr, heart; Kd, kidney; Ln, lung; Lv, liver; Ms, muscle; Sk, skin; Sp, spleen; wpc, weeks post conception.

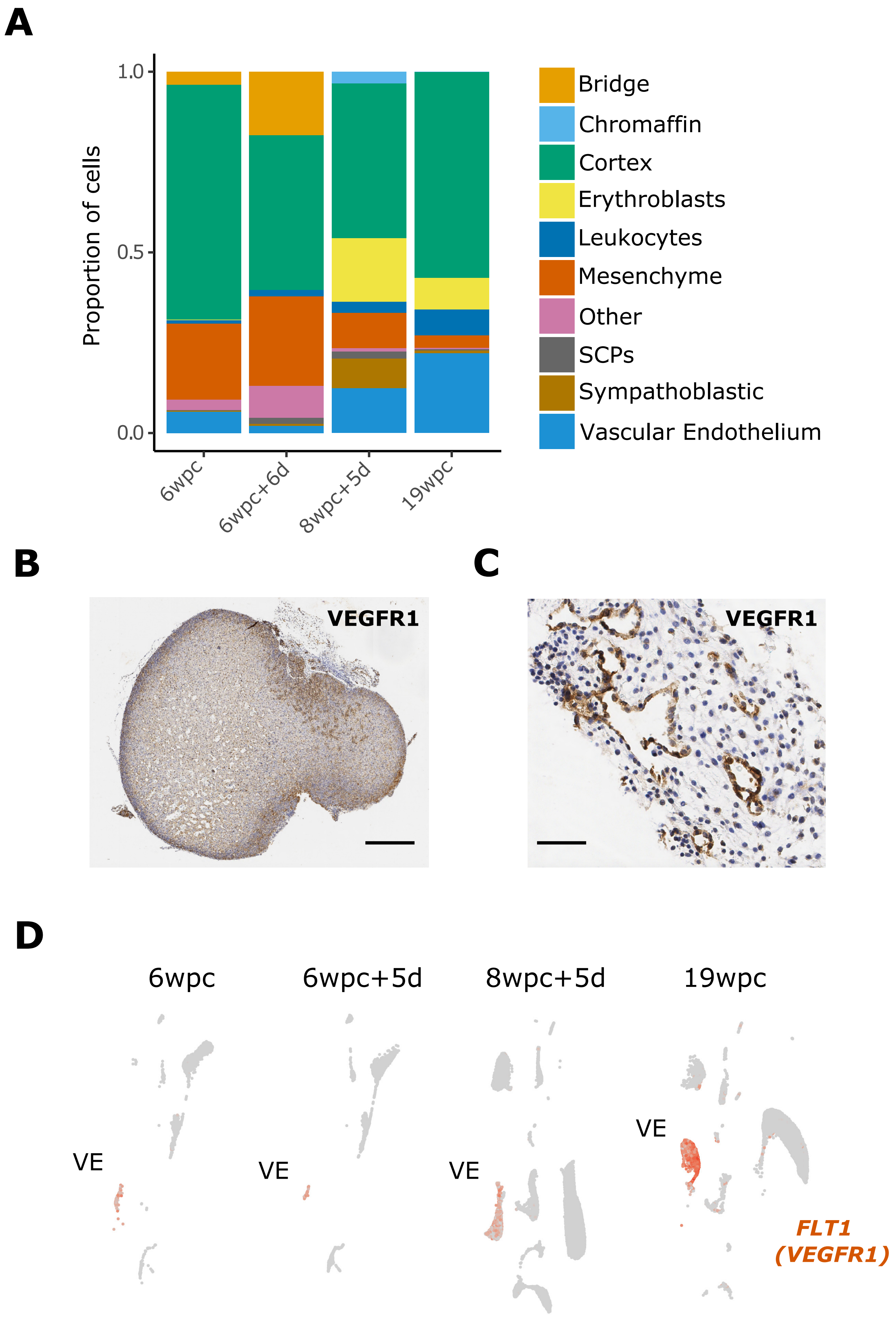

Supplemental Figure 3. Development of the vascular endothelium cluster and expression of (VEGFR1/*FLT1*). A Relative proportion of annotated clusters at each age. **B** Section of the adrenal gland at 8.5wpc stained for vascular endothelial growth factor receptor 1 (VEGFR1/FLT1) expression. Scale 400µm. **C** IHC of VEGFR1 (FLT1) expression in the vascular endothelium of surface blood vessels at 8.5wpc. Scale 50µm. **D** Feature plot of adrenal cell clusters at each stage showing vascular endothelial cells (VE) and changes in *FLT1* (*VEGFR1*) expression.

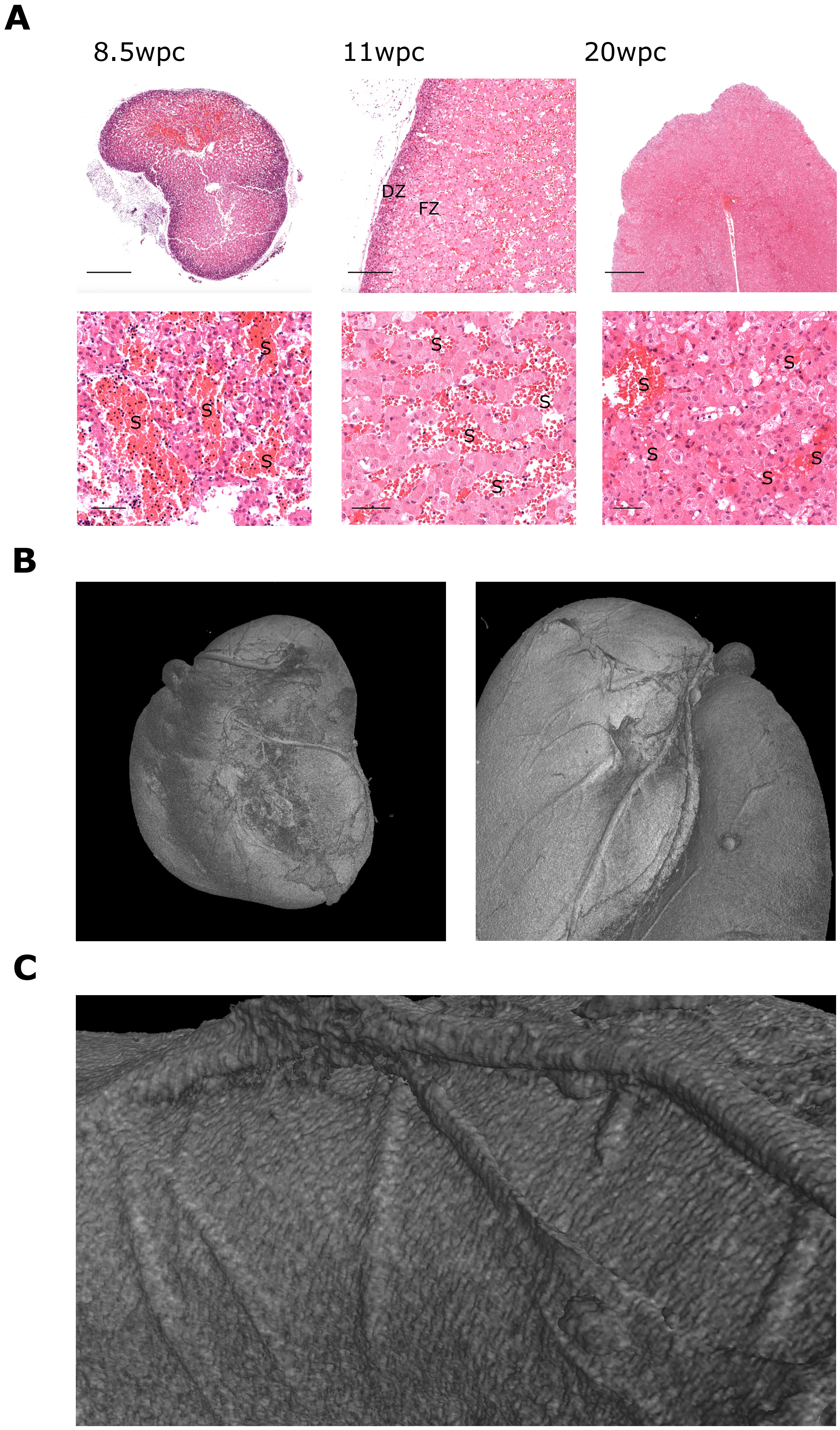

Supplemental Figure 4. Vascularization of the adrenal gland with development. A Histology of adrenal glands to show the developing network of vascular sinusoids (S) and central sinuses. Large fetal zone (FZ) cytomegalic cells are indicated, compared to the smaller definitive zone (DZ) cells with compact cytoplasm. Hematoxylin and eosin (H&E) staining. Scale bars: 8.5wpc, 400μm (upper), 50μm (lower); 11wpc, 200μm (upper), 50μm (lower); 20wpc, 800μm (upper), 50μm (lower). **B** MicroCT surface images showing vasculature at 17wpc. **C** MicroCT showing surface vessels branching and traversing the adrenal gland capsule at 17wpc.

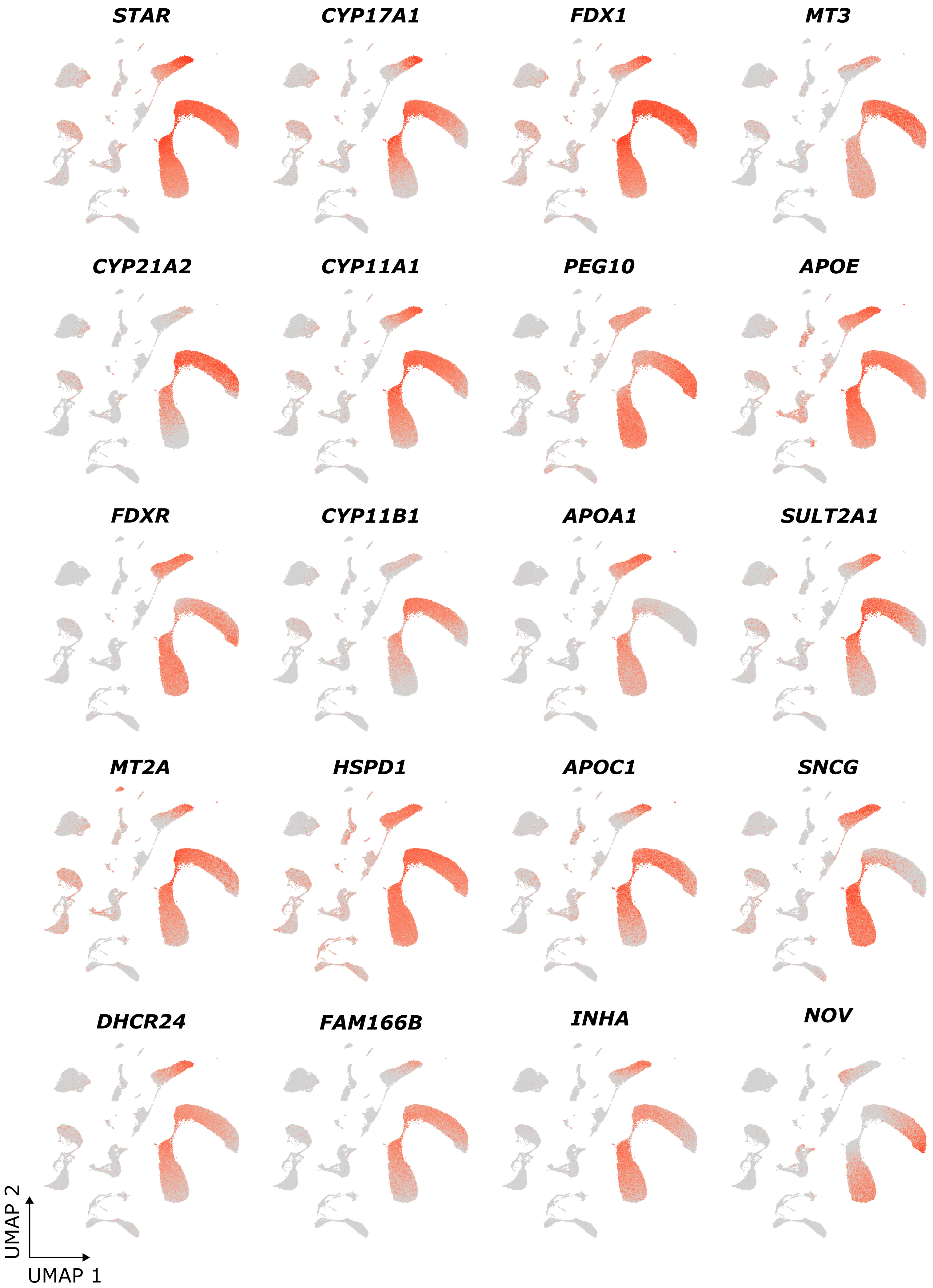

Supplemental Figure 5. Feature plots of the top differentially-expressed adrenal cortex cluster genes from the merged scRNA-seq dataset. The annotated clusters of the UMAP are shown in main Figure 1G and dot plot of expression of these genes is shown in main Figure 2A.

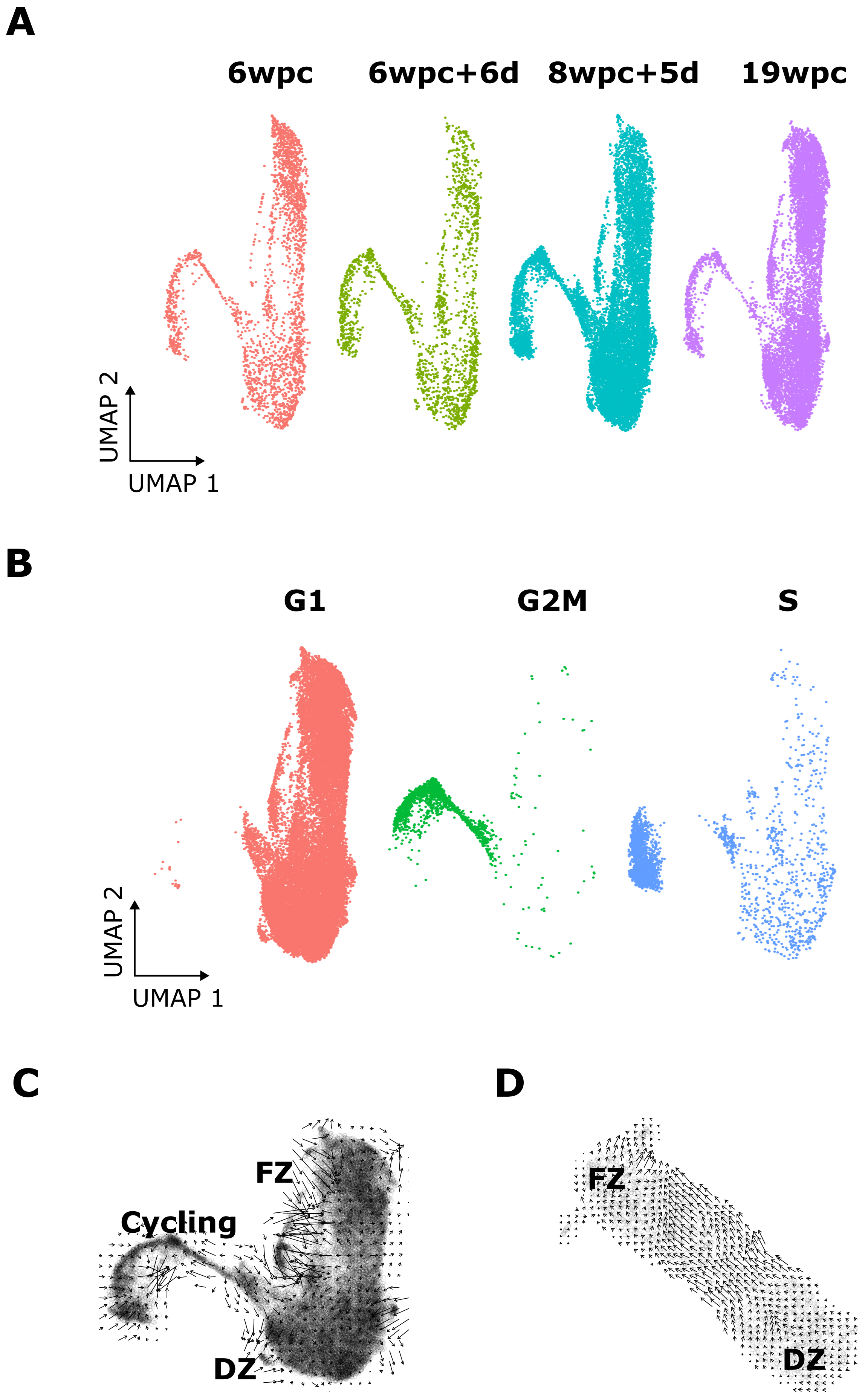

Supplemental Figure 6. Analysis of cycling cells and cell trajectories contributing to the integrated UMAP in main Fig. 2I. A Overview of the cortex and cortex dividing cells at each age. **B** Integrated UMAP showing cells in G1 phase (red) and dividing cells in GM2 phase (green) and S phase (blue). **C** RNA velocity showing cycling cell trajectories from a node closest to the definitive zone (DZ) rather than the fetal zone (FZ). **D** RNA velocity of the 6wpc scRNA-seq adrenal cortex cluster showing a potential trajectory from the DZ to FZ at this age.

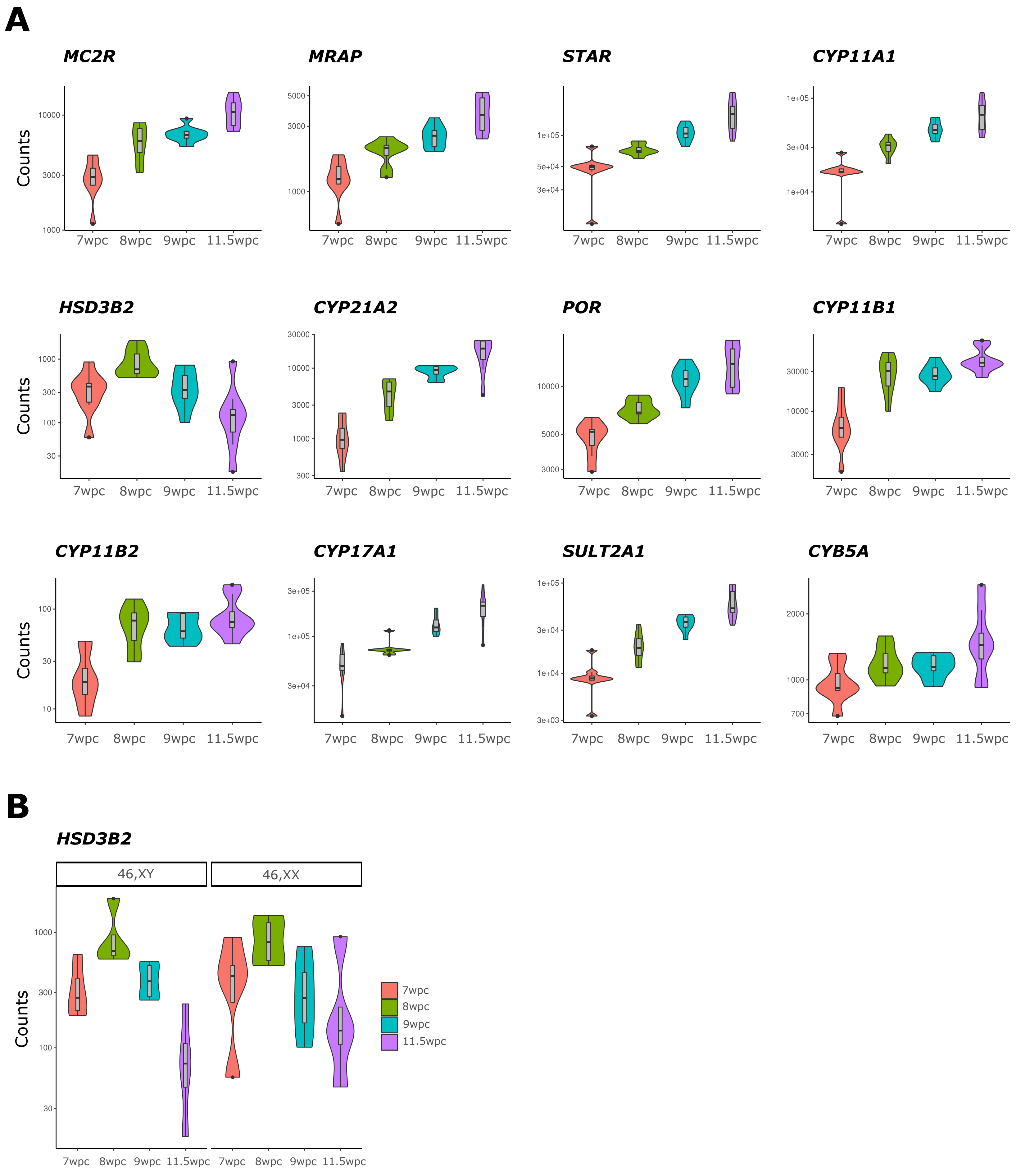

Supplemental Figure 7. Bulk RNA-seq time series changes in expression of key genes in the classic pathway of human adrenal steroidogenesis (see main Figure 3D for reference). A Data (normalized counts) are shown as violin plots at each of the four different ages analyzed (n=8 in each group). **B** Time-series expression of *HSD3B2* (normalized counts) in 46,XY (n=4 at each stage) and 46,XX (n=4 at each stage) samples.

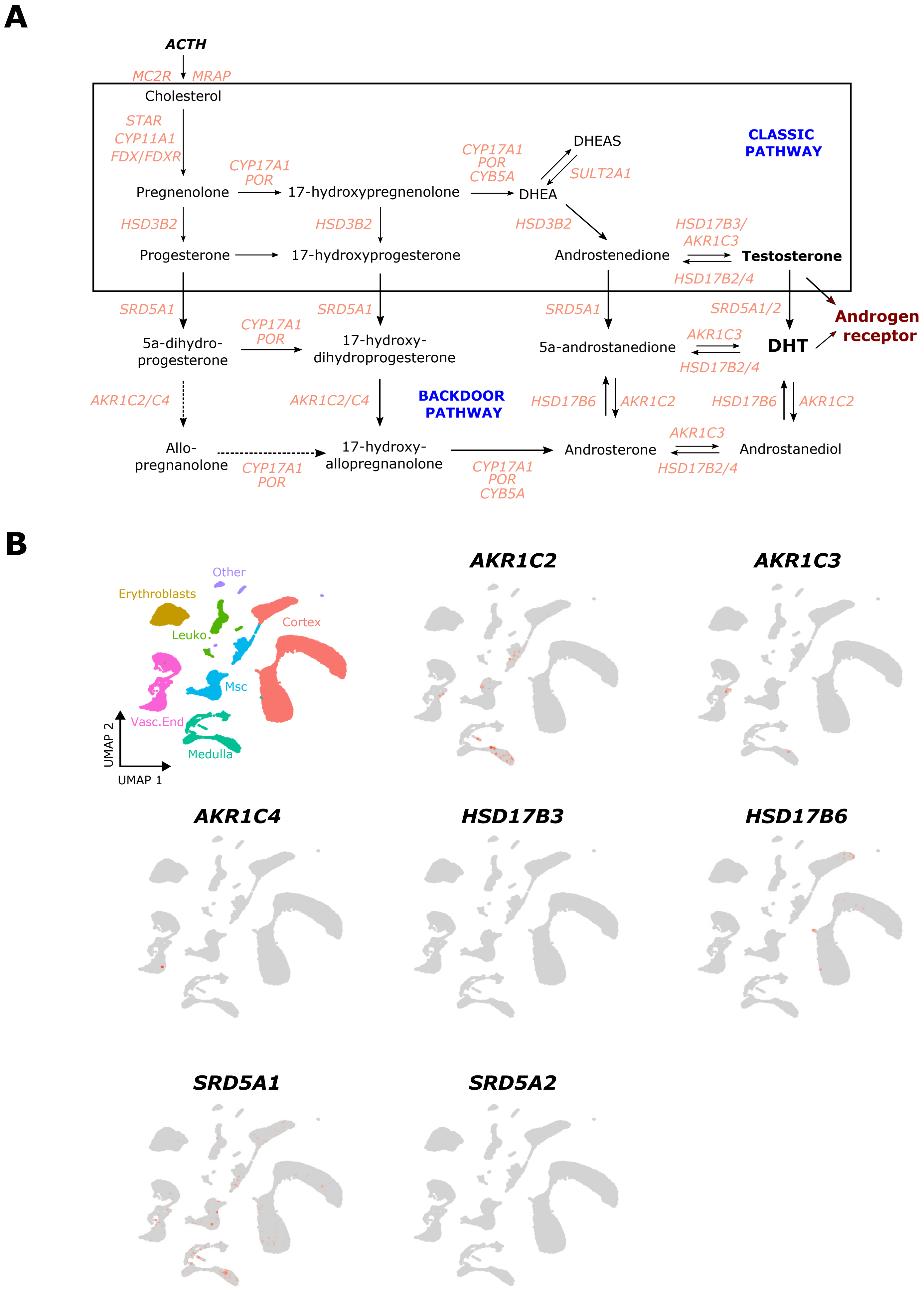

Supplemental Figure 8. Expression of “backdoor” pathway enzymes in the human fetal adrenal gland. A Overview of the potential “backdoor” pathway to androgen (dihyhdrotestosterone, DHT) synthesis. **B** Feature plots showing single-cell RNA-seq expression (merged datasets) for key components of the “backdoor” pathway. Msc, mesenchyme.

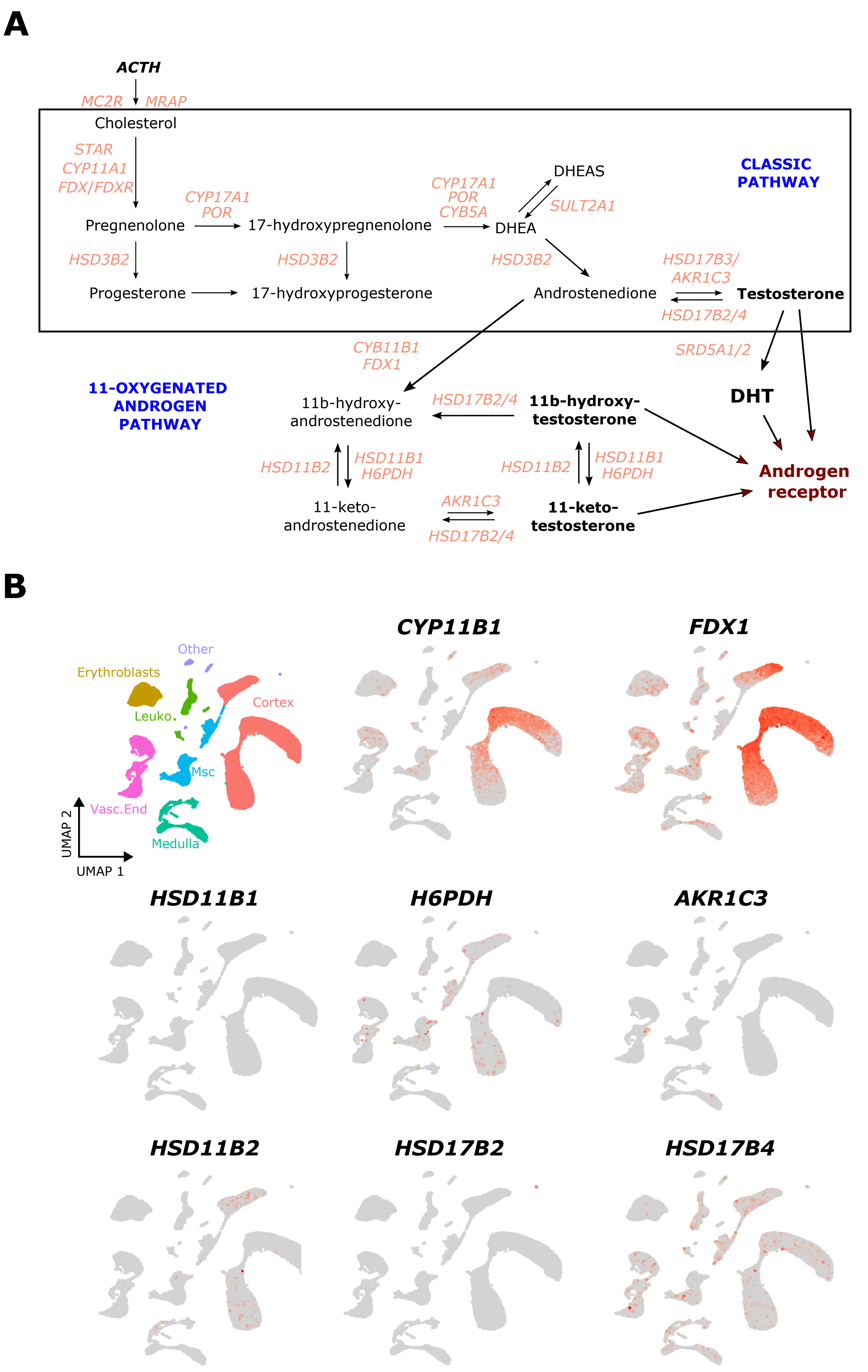

Supplemental Figure 9. Expression of enzymes needed for 11-oxygenation of androgens in the human fetal adrenal gland. A Overview of the potential "11-oxygenation" pathway. **B** Feature plots showing single-cell RNA-seq expression (merged dataset) for key components of the "11-oxygenation" pathway. Msc, mesenchyme.

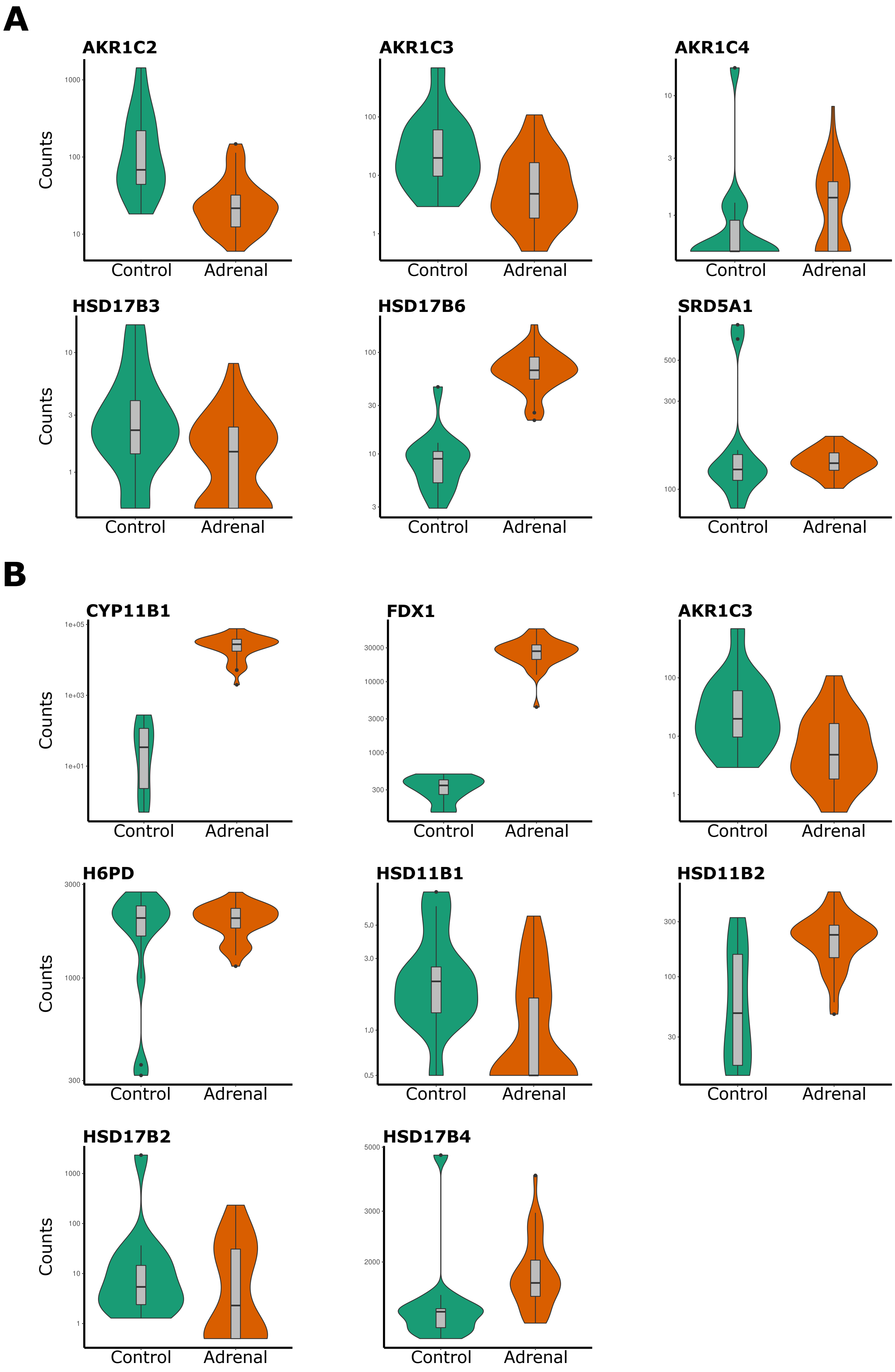

Supplemental Figure 10. Expression of alternative pathway enzymes in bulk RNA-seq data for the adrenal glands compared to controls. A Enzymes involved in the "backdoor" pathway. **B** Enzymes involved in 11-oxygenation of androgens. Data are shown as violin plots for normalized counts (control n=14, adrenal n=32).

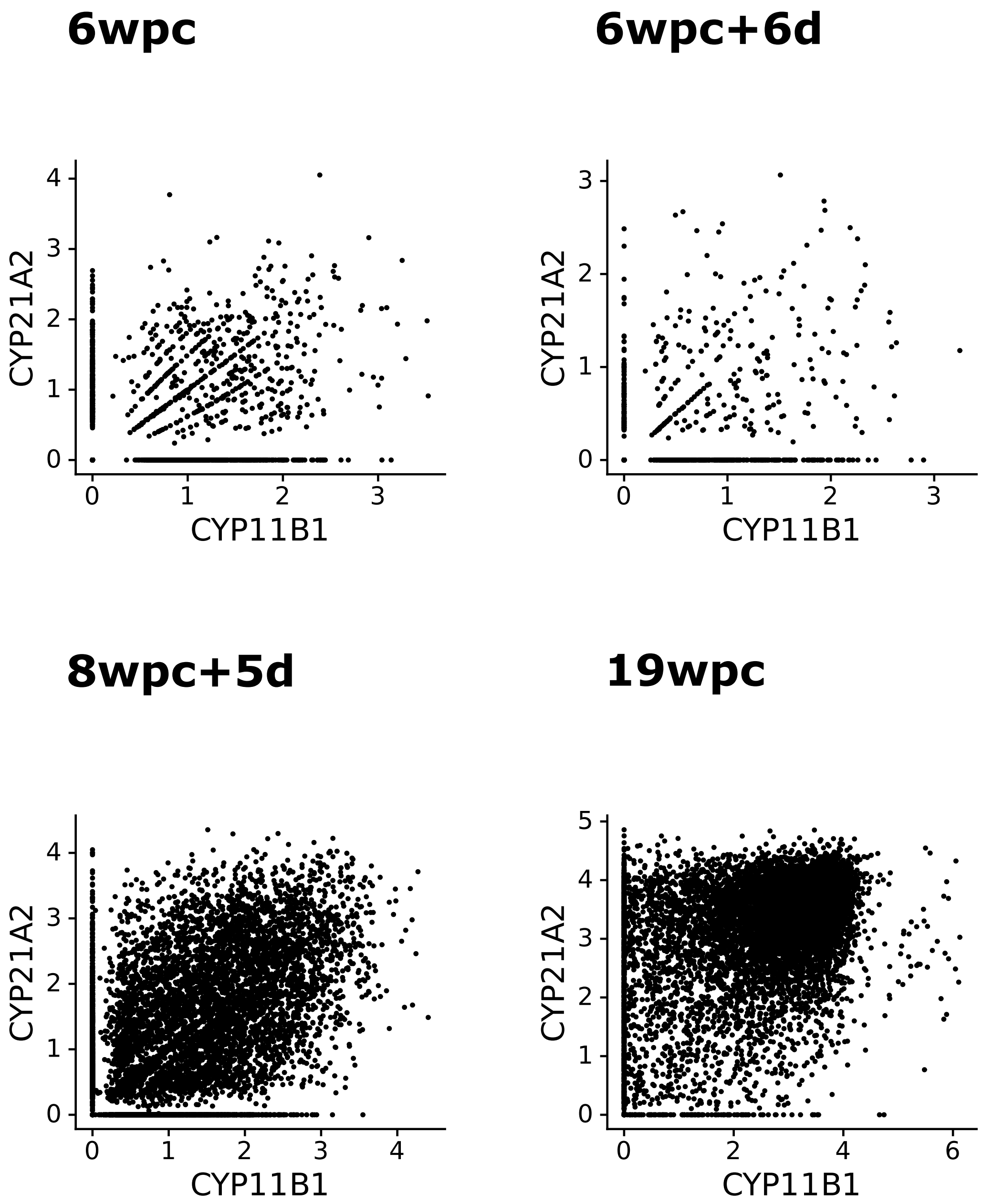

Supplemental Figure 11. Single-cell scatter plots showing expression of *CYP11B2* and *CYP21A2*. Data from all four ages are shown, demonstrating an increase in expression of both genes with time. Correlation Coefficients: 0.38 (6wpc), 0.37 (6wpc+6d), 0.59 (8wpc+5d) and 0.43 (19wpc). Note the different scales with each stage.

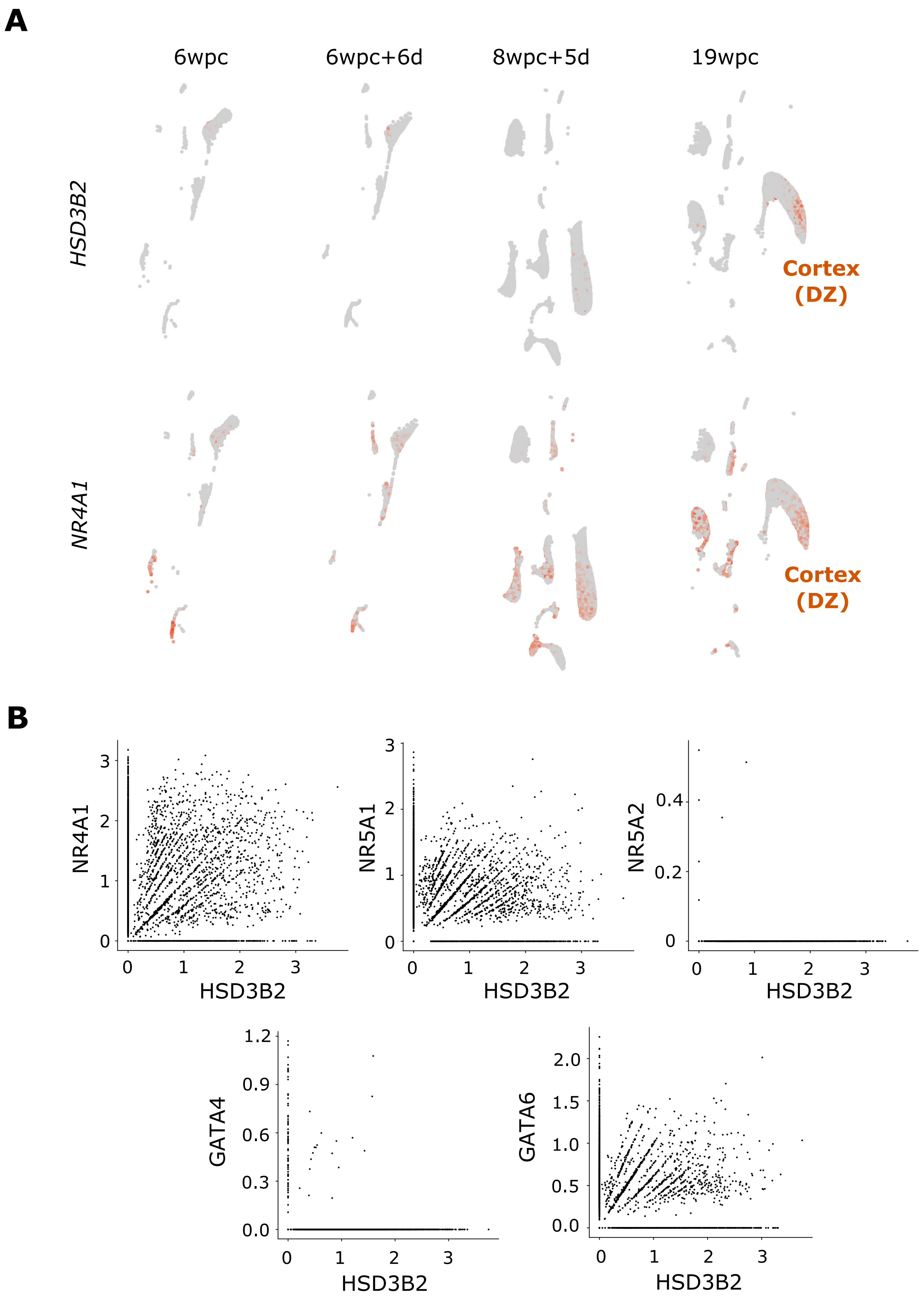

Supplemental Figure 12. Transcription factors associated with *HSD3B2* expression at 19wpc. A Feature plots of *HSD3B2* and *NR4A1* (*NURR77*). The adrenal cortex is shown with these genes expressed in the definitive/transition zone. **B** Single-cell scatter plots showing expression of *HSD3B2* and putative co-regulators. Data are shown at 19wpc for *NR4A1* (NURR77), *NR5A1* (SF-1), *NR5A2* (LRH-1), *GATA4* and *GATA6*.

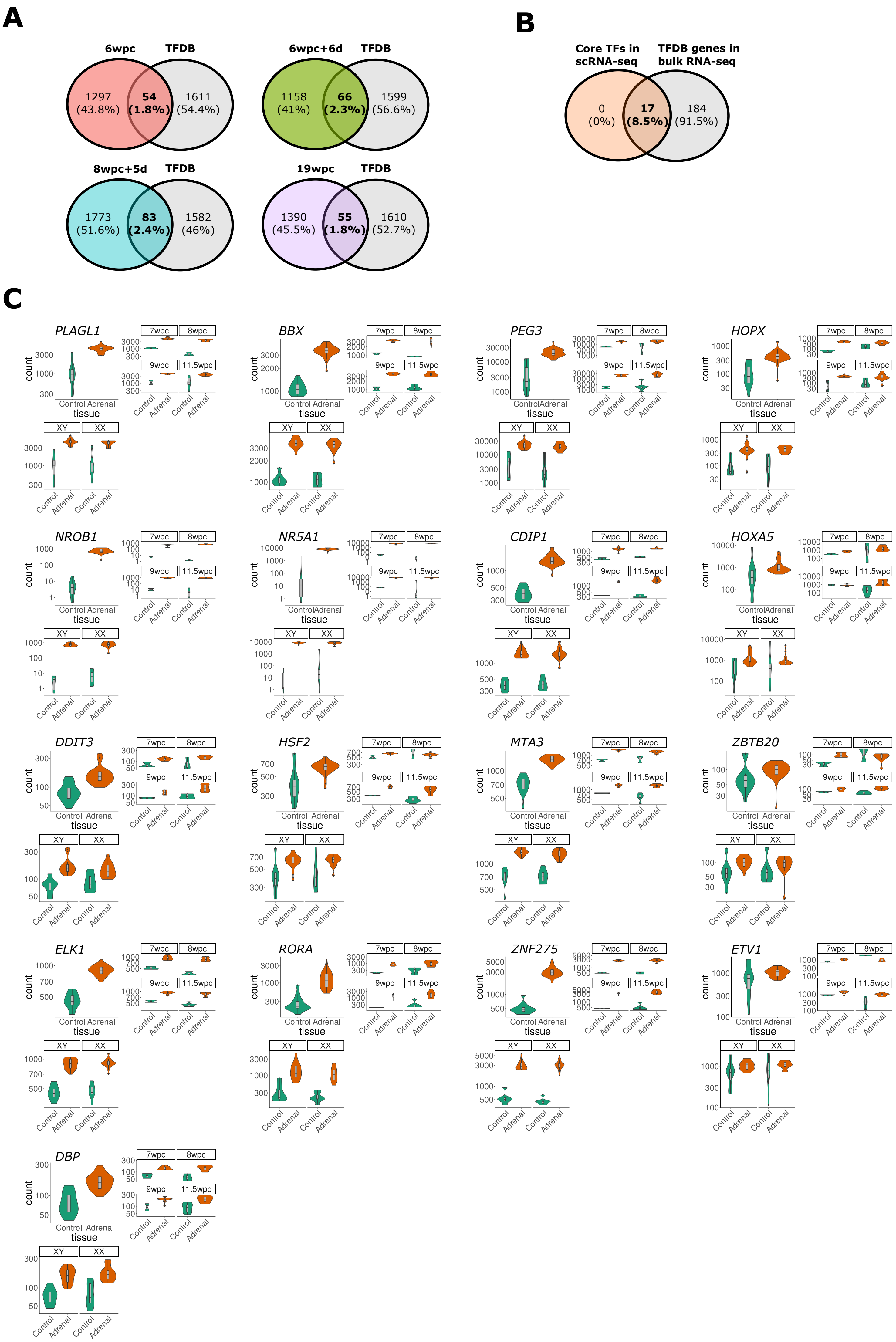
A
6wpc
TFDB
1297 (43.8%)
54 (1.8%)
1611 (54.4%)
6wpc+6d
TFDB
1158 (41%)
66 (2.3%)
1599 (56.6%)
8wpc+5d
TFDB
1773 (51.6%)
83 (2.4%)
1582 (46%)
19wpc
TFDB
1390 (45.5%)
55 (1.8%)
1610 (52.7%)
B
Core TFs in scRNA-seq
TFDB genes in bulk RNA-seq
0 (0%)
17 (8.5%)
184 (91.5%)
C
PLAGL1
BBX
PEG3
HOPX
NROB1
NR5A1
CDIP1
HOXA5
DDIT3
HSF2
MTA3
ZBTB20
ELK1
RORA
ZNF275
ETV1
DBP
count
tissue
Control
Adrenal
7wpc
8wpc
9wpc
11.5wpc
XY
XX

Supplemental Figure 13. Differentially-expressed transcription factors in the adrenal gland. **A** Venn diagrams of transcription factors (transcription factor database, TFDB) differentially-expressed in the single-cell RNA-seq data (cortex cluster) at each age (log2 fold-change >0.25, padj<0.05). **B** Venn diagram showing the distribution of these 17 core transcription factors (TFs) identified within the scRNA-seq dataset ("Core TFs in scRNA-seq") compared to TFDB genes that are differentially-expressed in the adrenal gland compared to controls using bulk-RNA seq ("TFDB bulk-RNA seq") (adrenal>control, padj<0.05). **C** Bulk RNA-seq normalized counts for core TFs showing adrenal expression (n=32) compared to controls (n=14) overall (*upper left individual panels*), expression compared to controls at each age (n=8 each stage) (*upper right individual panels*) and expression in 46,XY and 46,XX samples (*lower left individual panels*).

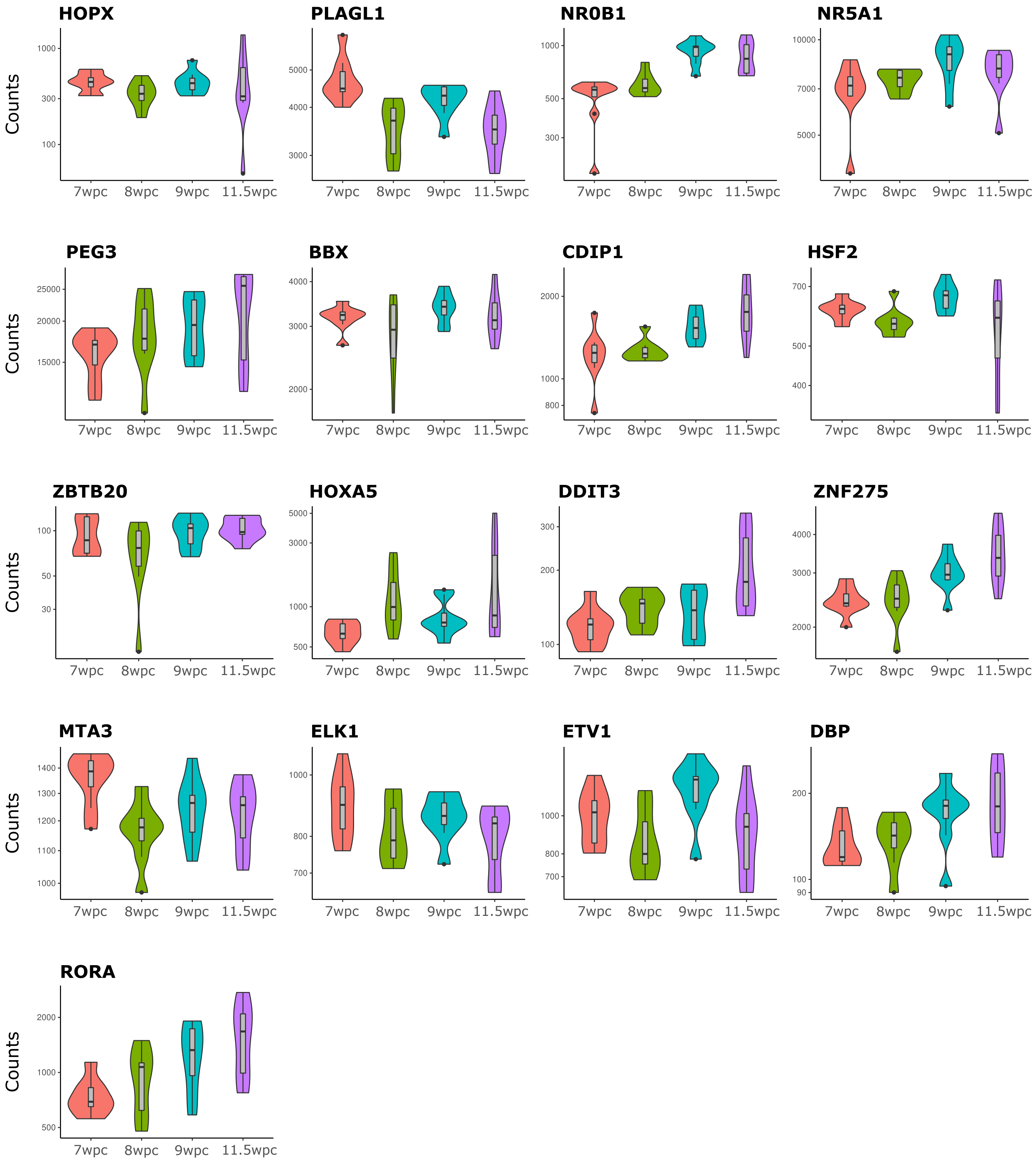

Supplemental Figure 14. Bulk RNA-seq of “core” transcription factor expression at each age. Data are shown as violin plots of normalized counts (each stage, n=8).

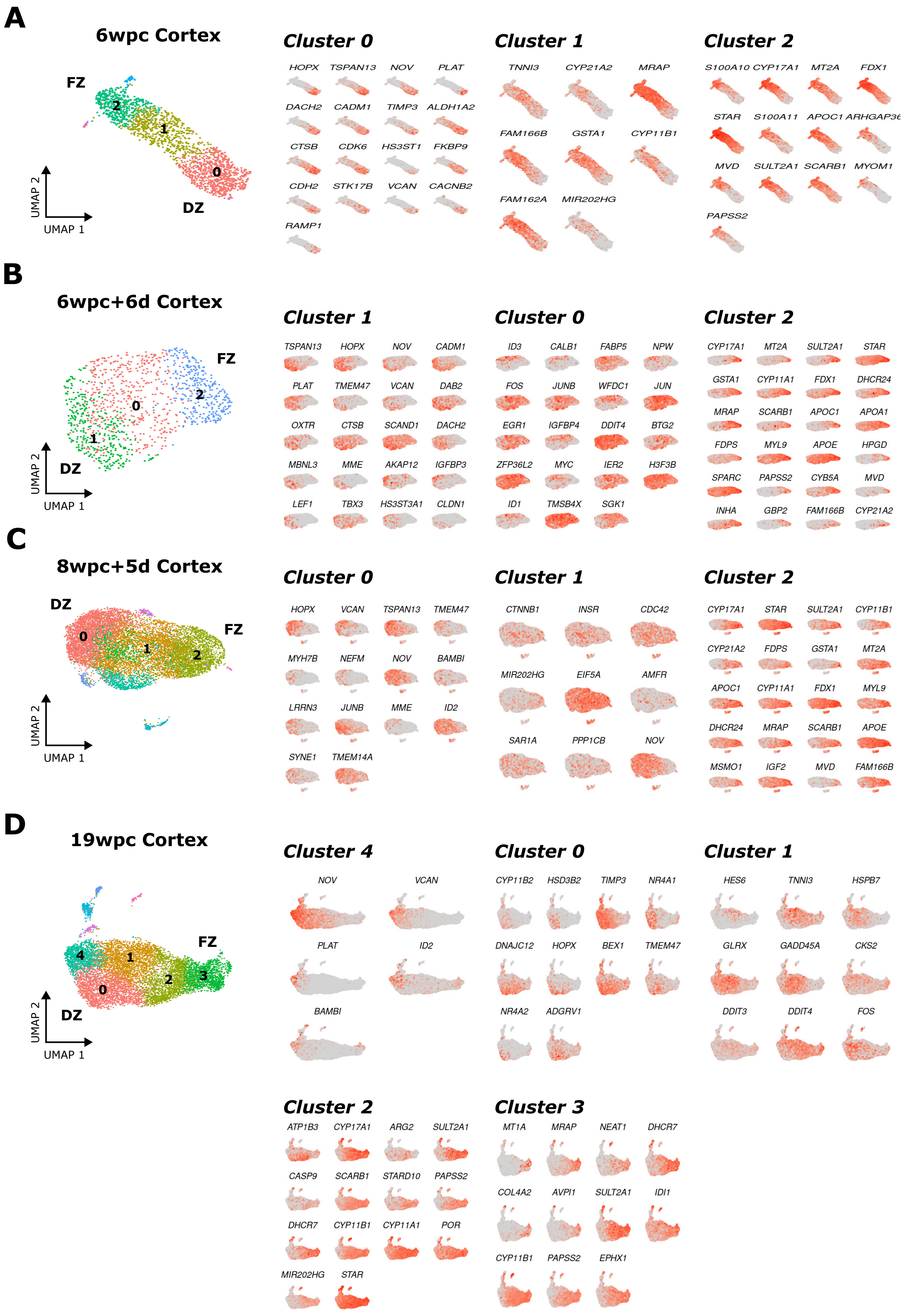

Supplemental Figure 15. UMAP and feature plots for scRNA-seq data showing the key differentially-expressed genes in each cluster identified at each age. A 6wpc. **B** 6wpc+6d. **C** 8wpc+5d. **D** 19wpc**.** DZ, definitive zone; FZ, fetal zone. (See Supplementary Data 3 for complete list of gene expression data at each age).

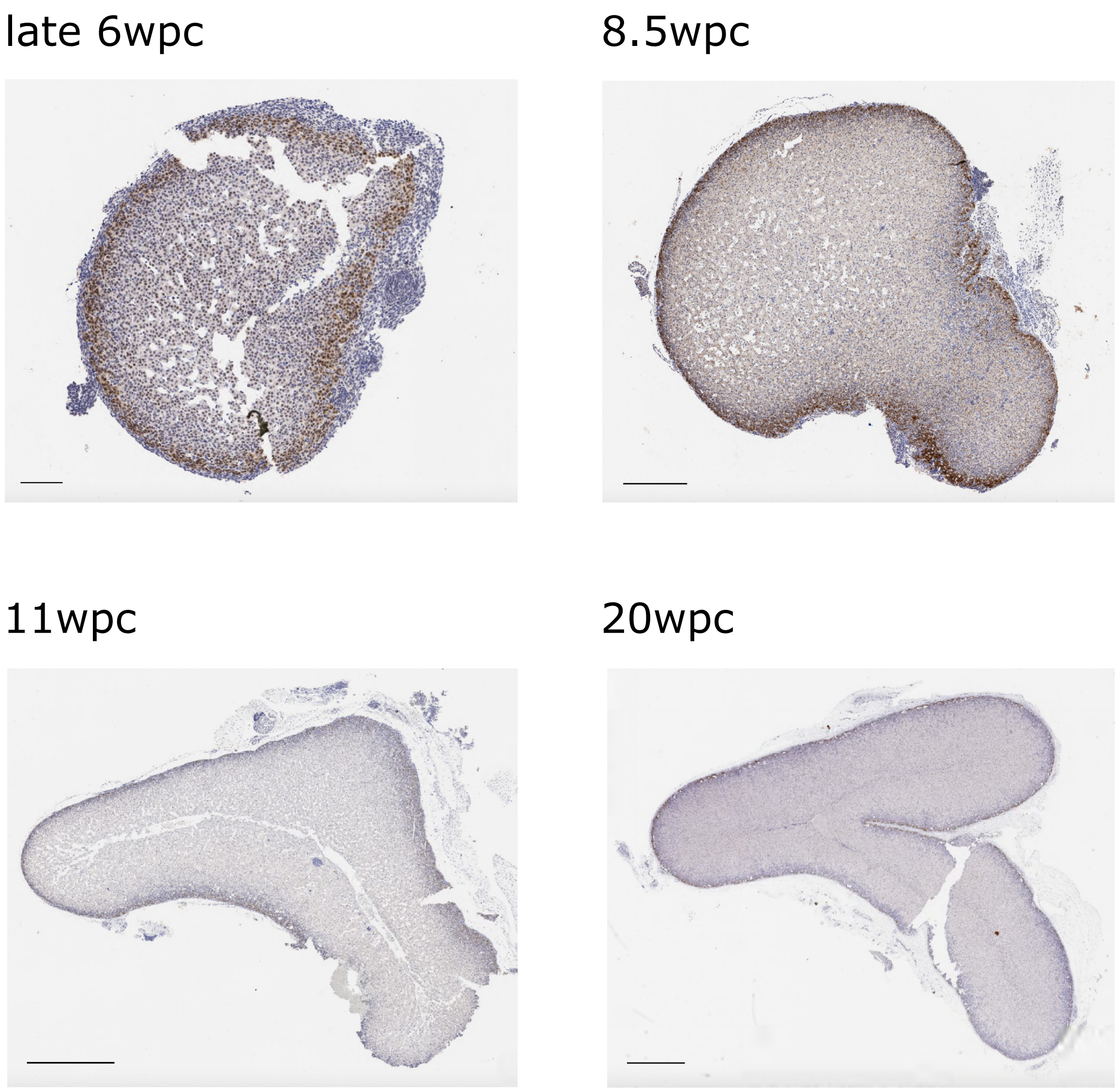

Supplemental Figure 16. Immunohistochemistry of HOPX in the human adrenal gland at different ages. Localized higher-power images are shown in Figure 5E. Scale bars: late 6wpc, 100µm; 8.5wpc, 400µm; 11wpc, 800µm; 20wpc, 1000µm.

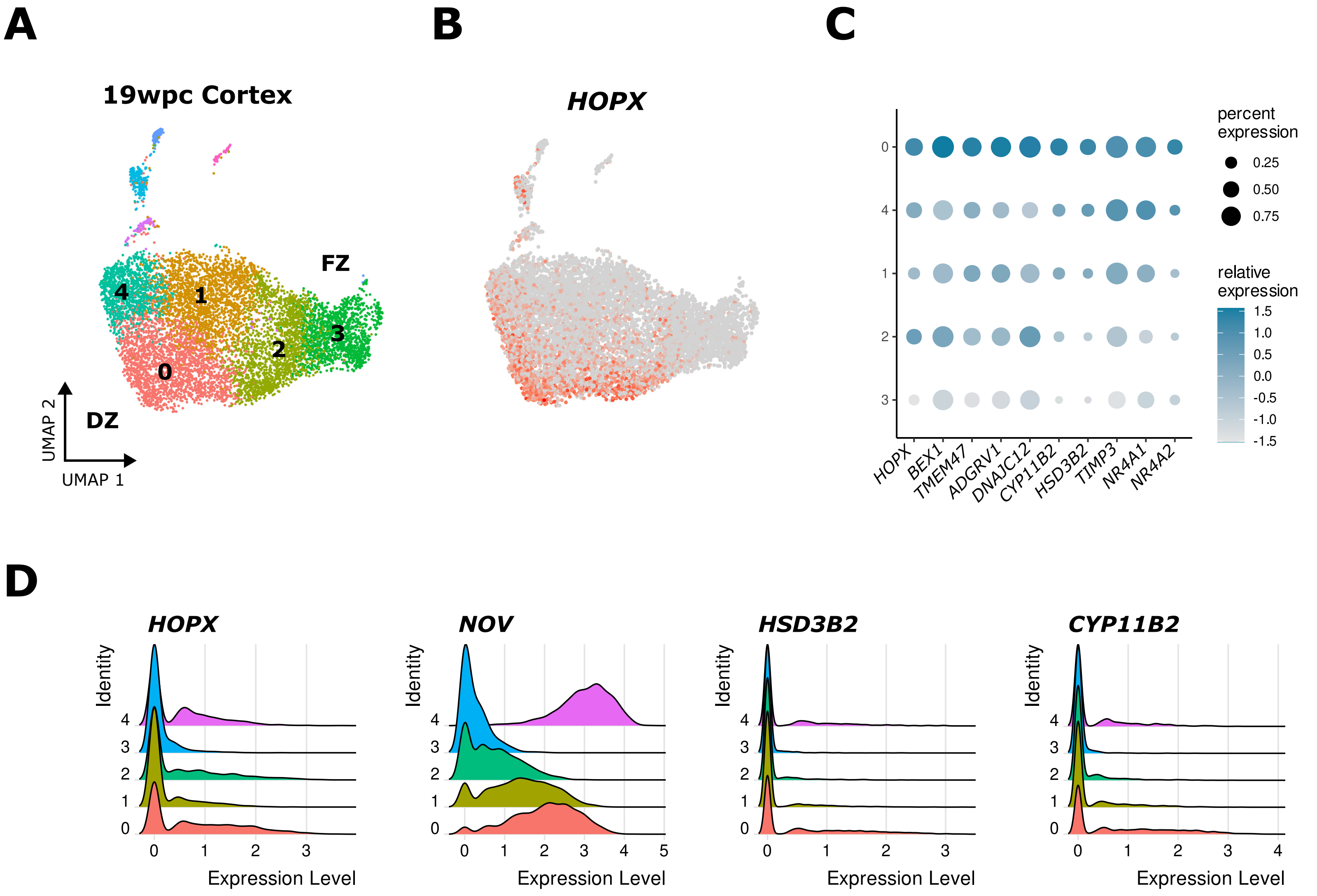

Supplemental Figure 17. Co-expression of *HOPX*/HOPX with related genes of interest in a 19wpc adrenal gland. A UMAP of key clusters in scRNA-seq data. **B** Feature plot of *HOPX* expression. **C** Dot plot showing relative expression of the top 10 differentially-expressed genes in cluster 0 compared to other clusters (1, 2, 3, 4). **D** Ridge plots of *HOPX* expression and related genes of interest in cluster 0 and cluster 4.

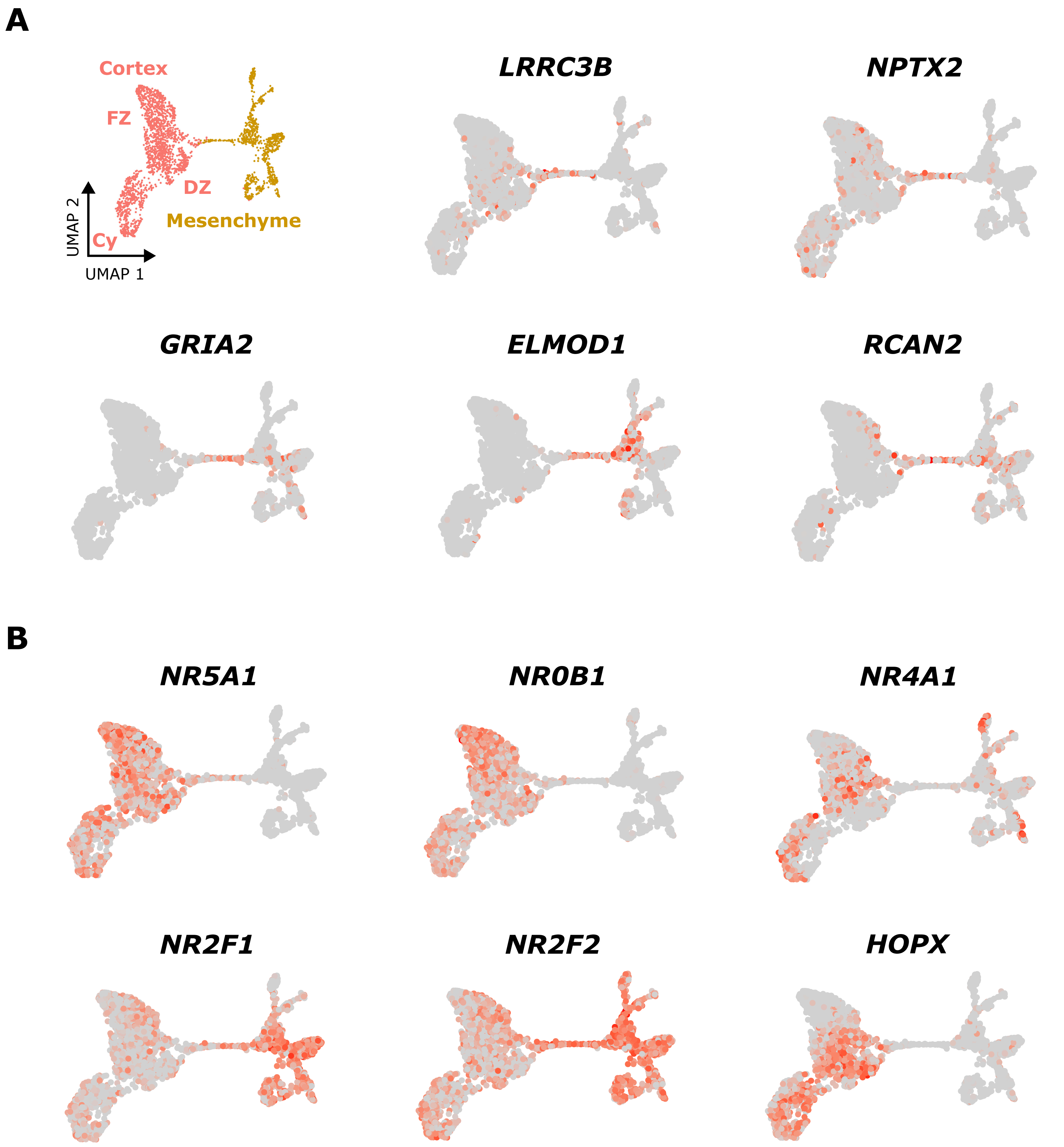

Supplemental Figure 18. Expression of key genes expressed across the mesenchyme and adrenal cortex clusters at 6wpc+6d. A Reference UMAP showing mesenchyme and adrenal cortex clusters as well has genes with high specificity for the "bridge" cluster. "High specificity" defined as padj <0.05, log2FC>0.5, Pct.2<0.2, see Supplementary Data 6. **B** Feature plot showing expression of key nuclear receptor transcription factors between the mesenchyme and adrenal cortex, as well as *HOPX*. Cy, cycling cells; DZ, definitive zone; FZ, fetal zone.

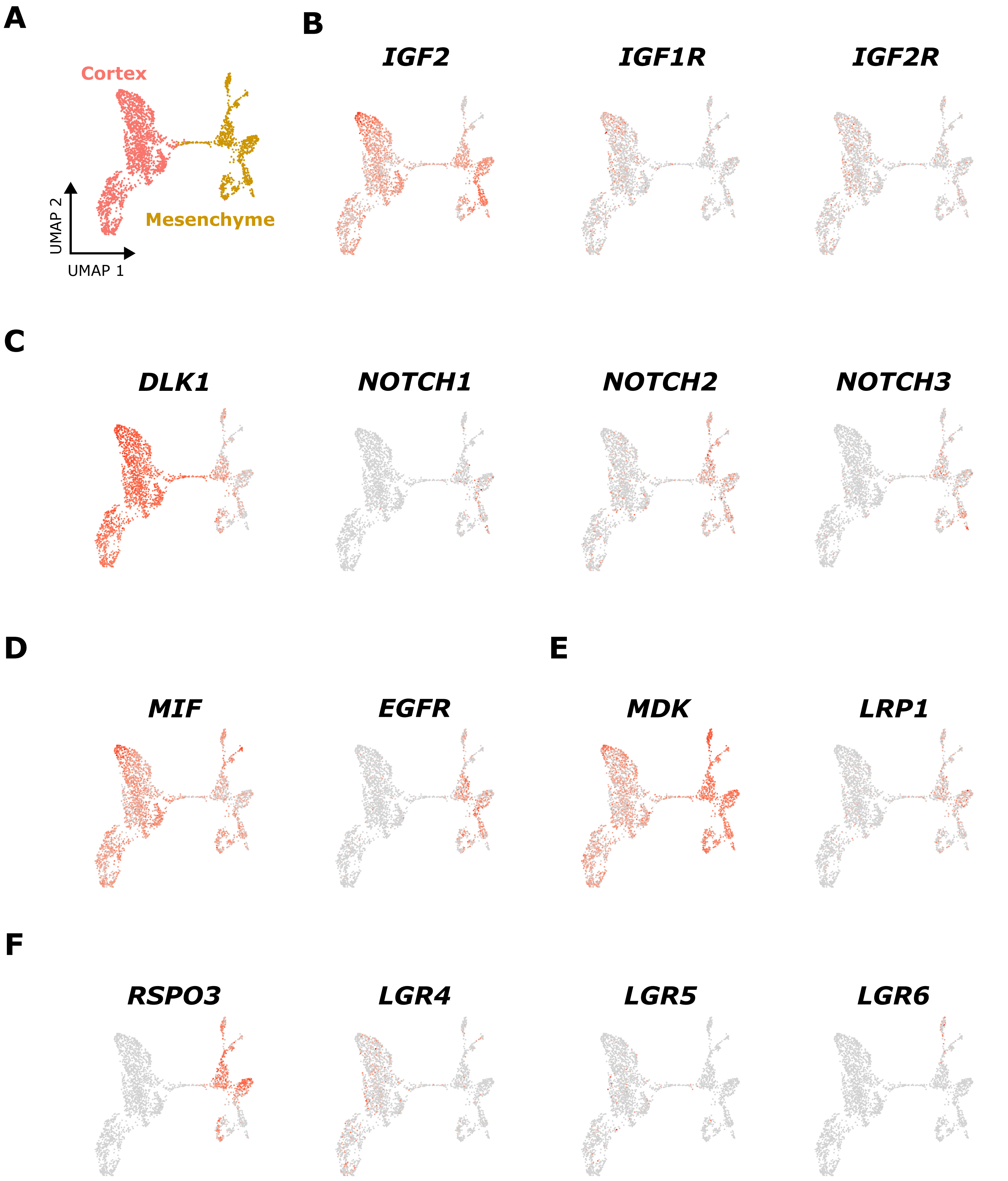

Supplemental Figure 19. Expression of key genes involved in ligand-receptor interactions in the mesenchyme and adrenal cortex clusters at 6wpc+6d identified by cell-cell communication network analysis. Data relates to main Fig. 6b. **A** Reference UMAP showing mesenchyme and adrenal cortex clusters. **B** Feature plot showing expression of *IGF2* (ligand) and related cognate receptors. **C** *DLK1* (ligand) and *Notch* family receptors. **D** *MIF* (ligand) and *EGFR*. **E** *MDK* (ligand) and *LRP1*. **F** *RSPO3* (ligand) and related *LGR* receptors.

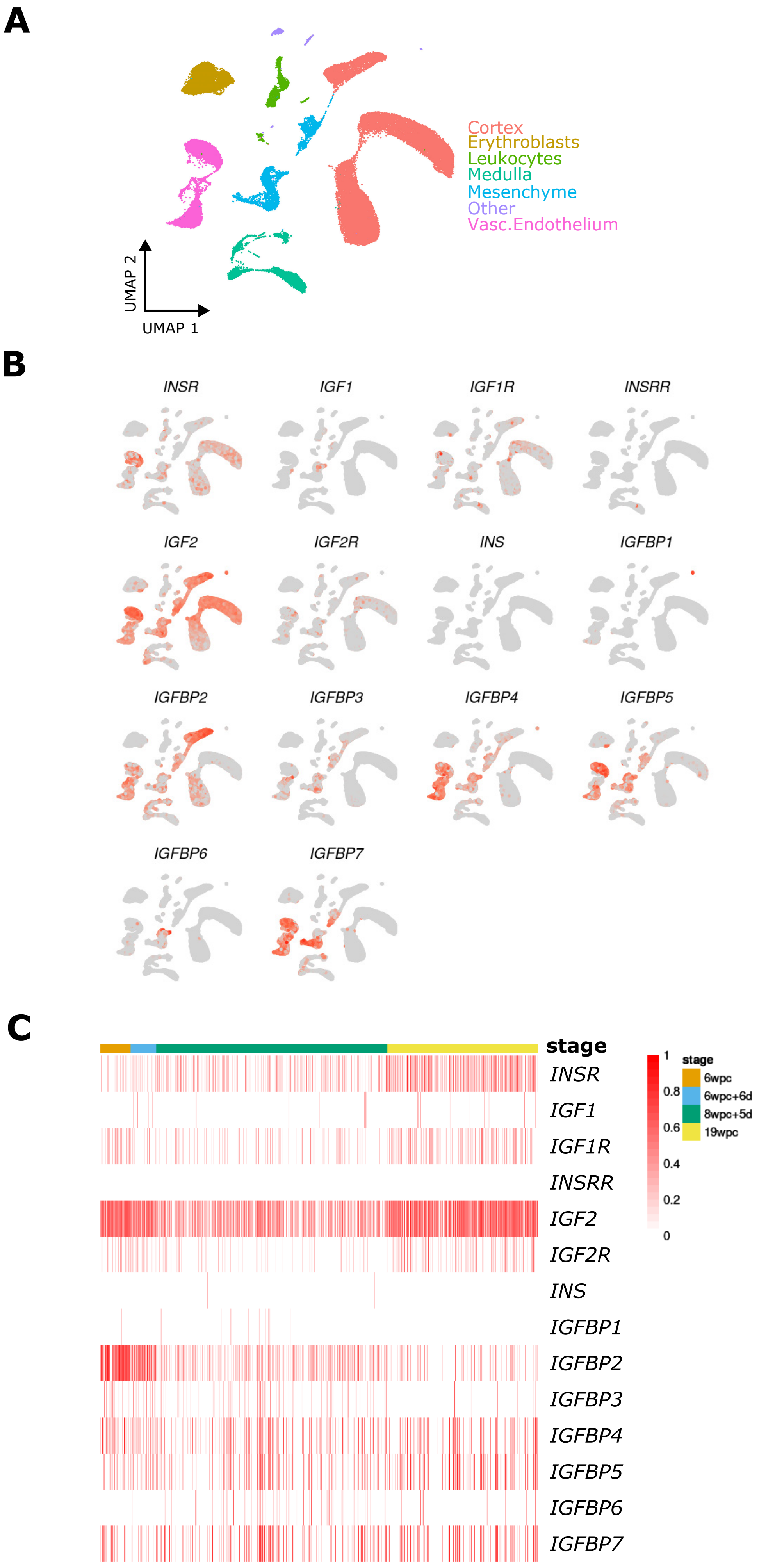

Supplemental Figure 20. scRNA-seq expression of insulin-like growth factor II (*IGF2*) and related genes in the adrenal gland. A Reference merged UMAP (as shown in Fig. 1g). **B** Feature plot for all clusters in the adrenal gland showing expression of *IGF2* and related genes. **C** Heatmap of expression of *IGF2* and related genes in the adrenal cortex cluster across the four ages studied.

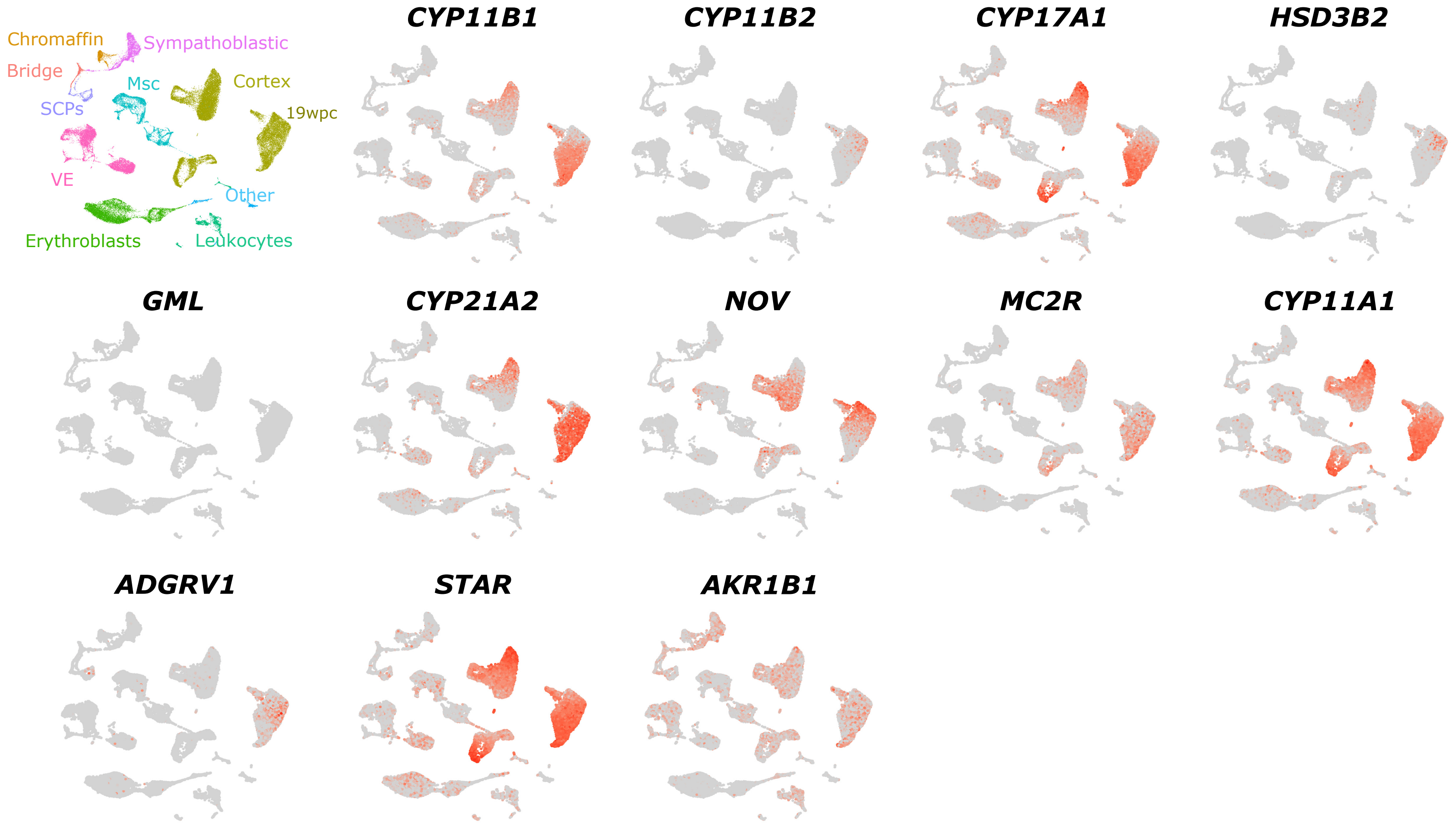

Supplemental Figure 21. Feature plots showing fetal adrenal expression of genes corresponding to the genes (n=12) that are most highly differentially expressed in the adult adrenal gland (Human Protein Atlas, www.proteinatlas.org). Data refer to the dot plot in Main Figure 8A, but show the increase in expression of several genes with age (19wpc cluster indicated).

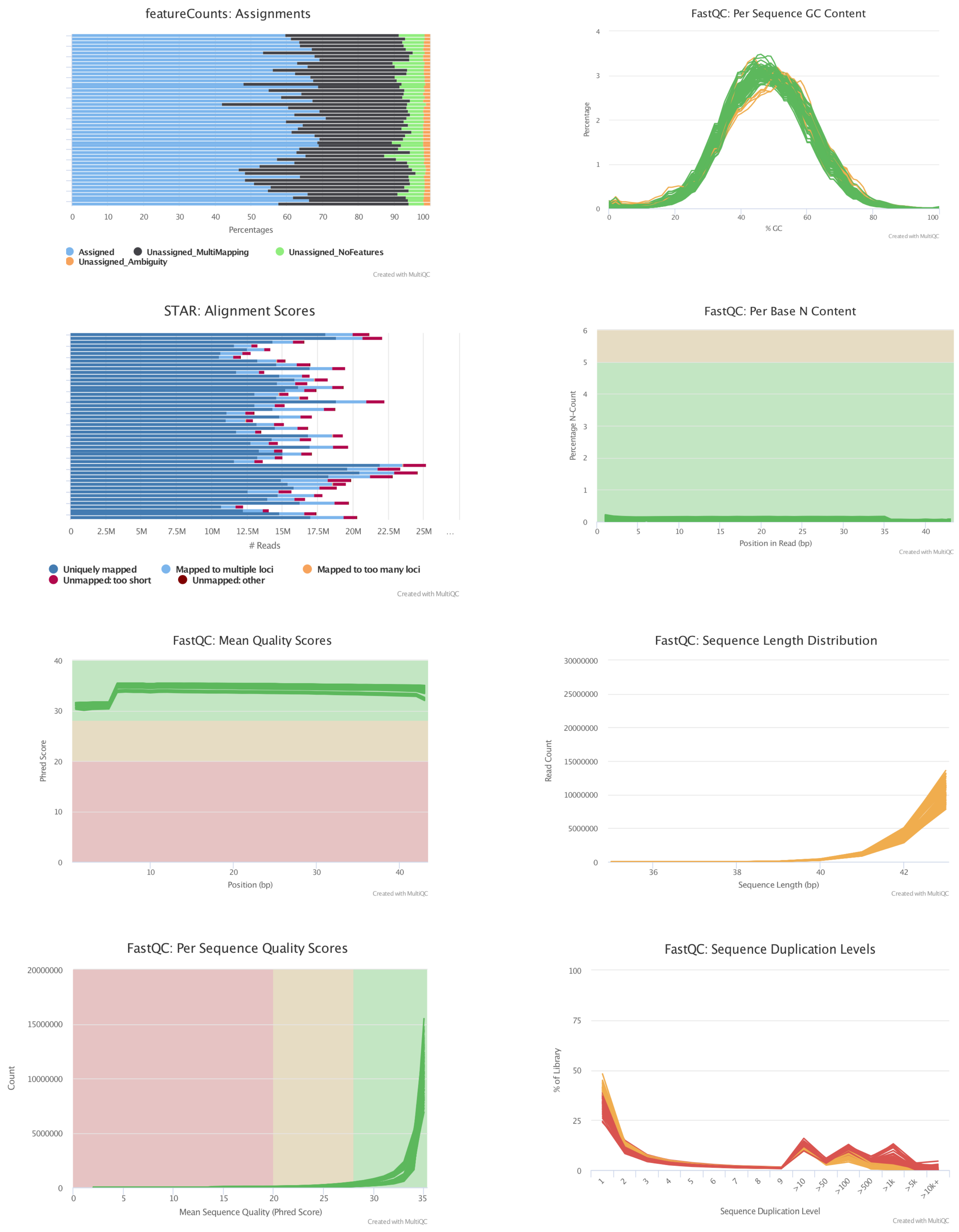

Supplemental Figure 22. MultiQC analysis for the bulk RNA-seq dataset.
